# Supplementary material for: Active learning of the thermodynamics-dynamics trade-off in protein condensates
Source: Sci Adv. 2024 Jan 5;10(1):eadj2448. doi: 10.1126/sciadv.adj2448 (PMC10775998; doi:10.1126/sciadv.adj2448)
Supplement: Supplementary file 1 — Supplementary Text Figs. S1 to S12 Tables S1 to S6 References [file sciadv.adj2448_sm.pdf]

Science Advances
AAAS

Supplementary Materials for

Active learning of the thermodynamics-dynamics trade-off in protein condensates

Yaxin An *et al.*

Corresponding author: Michael A. Webb, mawebb@princeton.edu; William M. Jacobs, wjacobs@princeton.edu

Sci. Adv. **10**, eadj2448 (2024)
DOI: 10.1126/sciadv.adj2448

This PDF file includes:

Supplementary Text
Figs. S1 to S12
Tables S1 to S6
References

1 Extended methods

1.1 Featurization of protein sequences

A 30-dimensional feature vector $\vec{x}$ is used as a numerical encoding of a polypeptide sequence and its chemical characteristics. To construct this feature vector, 30 different characteristics of each protein sequence are considered, including

- the composition of each amino acid in the sequence, c_a, where a corresponds to one of the amino acids (A, C, D, E, F, G, H, I, K, L, M, N, P, Q, R, S, T, V, W, and Y) and $\sum_a c_a = 1$;
- the sequence length (i.e., number of amino acids), N;
- the net charge per residue, $|\bar{q}|$,
$$|\bar{q}| \equiv \frac{1}{N}\left|\sum_{i=1}^{N} q_i\right|;$$
- the sequence charge decoration, SCD,
$$\text{SCD} \equiv \frac{1}{N}\sum_{i=1}^{N}\sum_{j=i+1}^{N} q_i q_j (j-i)^{1/2};$$
- the average hydrophobicity per residue, $\bar{\lambda}$,
$$\bar{\lambda} \equiv \frac{1}{N}\sum_{i=1}^{N} \lambda_i;$$
- the sequence hydropathy decoration, SHD,
$$\text{SHD} \equiv \frac{1}{N}\sum_{i=1}^{N}\sum_{j=i+1}^{N} (\lambda_i + \lambda_j)(j-i)^{-1};$$
- a mean-field prediction of the second-virial coefficient, $B_2^{(\text{MF})}$,
$$B_2^{(\text{MF})} \equiv \sum_{i}^{N}\sum_{j}^{N} b_{2,ij},$$
where $b_{2,ij} \equiv 2\pi \int_0^\infty dr\, r^2 \left[1 - e^{-\beta u_{ij}(r)}\right]$ is the second-virial coefficient between monomers i and j, which are assumed to be on different chains, and $u_{ij}(r)$ is the pair potential between monomers i and j;

- the fraction of positively charged residues, $\bar{q}_+$;
- the fraction of negatively charged residues, $\bar{q}_-$;
- the Shannon entropy, S,

$$S \equiv -\sum_a c_a \log c_a;$$

- and the average molar mass of a residue, $\bar{M}$,

$$\bar{M} \equiv \frac{1}{N}\sum_{i=1}^{N} M_i,$$

  where M_i is the molecular weight of the ith amino acid in the sequence.

For the purpose of using these sequence characteristics as features in machine learning and optimization, it is useful to transform the variables such that they have similar magnitudes. Therefore, we apply normalization techniques to each of the last ten sequence characteristics (i.e., all features except the $\{c_a\}$, which are already on a unit scale). With the exception of N and S, standard normalization is used for each feature:

$$\tilde{A} = \frac{A - \mu_A}{\sigma_A}, \tag{1}$$

where A is a given sequence characteristic with an average of μ_A and a standard deviation of σ_A estimated over the sequences that comprise the current dataset $\mathcal{D}$ for training the machine-learning models. Min-max normalization is employed for N,

$$\tilde{N} = \frac{N - \min_{\mathcal{D}} N}{\max_{\mathcal{D}} N - \min_{\mathcal{D}} N}, \tag{2}$$

and normalization by the maximum is employed for S,

$$\tilde{S} = \frac{S}{\max_{\mathcal{D}} S} - 1. \tag{3}$$

We note that similar feature vectors have demonstrated excellent predictive capabilities relating to both structural and dynamic properties of intrinsically disordered protein chains (58,59).

1.2 Gaussian process regression models

Gaussian process regression (GPR) is used to estimate expected values and uncertainties for the second-virial coefficient, B_2, and the condensed-phase self-diffusion coefficient, D, as a function of the feature vector, $\vec{x}$. GPR is particularly used for its native uncertainty estimates, which are central to the computation of expected hypervolume improvement (EHVI) acquisition

function described in the main text, Eq. (4). Separate GPR models are constructed for B_2 and D. Covariances modeled by the Gaussian processes are calculated using a Matérn kernel ($\nu = 3/2$) with added noise,

$$k(\vec{x}, \vec{x}') = \sigma^2 \left[\frac{1}{\Gamma(3/2)2^{1/2}} \times \left(\frac{\sqrt{3}}{l} ||\vec{x} - \vec{x}'||_2 \right)^{3/2} K_{3/2} \left(\frac{\sqrt{3}}{l} ||\vec{x} - \vec{x}'||_2 \right) \right] + \sigma_n^2. \quad (4)$$

In Eq. (4), σ, l, and σ_n are treated as adjustable hyperparameters, $\Gamma(\cdot)$ denotes the gamma function, and $K_{3/2}$ is a modified Bessel function. Hyperparamater optimization is predicated on minimization of the mean-squared errors. Five-fold cross-validation is employed on the training set to mitigate overfitting and obtain optimal hyperparameters. In particular, five sets of optimized hyperparameters are produced (one for each fold), and these sets are then averaged to yield a final set of parameters. Using this final set, a GPR model is trained over the entirety of the data acquired up to that point for the purpose of surrogate modeling during sequence optimization. The performance of the GPR models at the conclusion of active learning for an 80/20 train/test split is shown in Fig. S6a,b and illustrates good predictive ability.

1.3 Random forest classifier construction

A random forest (RF) classifier is used to predict whether phase separation is expected to occur as a function of the feature vector, $\vec{x}$. Here, the RF classifier consists of 100 decision trees. The best split in decision trees is determined by the Gini impurity. The minimum number of samples required to split an internal node is set at two, and the minimum number of samples required to be a leaf node is one. Five-fold cross-validation is employed on the training set to mitigate overfitting and to obtain optimal hyperparameters in the same fashion as described in SI Sec. 1.2. The performance of the RF classifier at the conclusion of active learning for an 80/20 train/test split is shown as a confusion matrix in Fig. S6c.

1.4 Sequence optimization and genetic algorithm

Sequence optimization to identify candidate proteins with high EHVI is facilitated using a genetic algorithm. In addition to conventional mutation and crossover moves, we also employ “deletion” and “growth” moves to improve diversity of proposed sequences. All moves are constrained as necessary to produce sequences with $20 \leq N \leq 50$. In all discussion, sequences of N residues are index from 1 to N. In each iteration, batches of 96 candidate sequences are produced as described below.

- *Step 1*: A set of n possible parent sequences sorted by their fitness from high to low. In Iteration 0, the parent sequences are sequences from DisProt that exhibit negative B_2. At the beginning of every other iteration, the parent sequences correspond to those sequences that define the current approximation to the Pareto front.

- *Step 2*: To generate "child" sequences, a pair of sequences are randomly selected from the top 30% of parent sequences. These sequences then undergo a series of crossover, mutation, growth and deletion moves:

  - *Crossover*: Suppose the pair of selected sequences have a length of N and M. First, two indices are selected s_1 and s_2 both from the set $\in [1, \min(\{N, M\})$ with uniform probability. Then, another index is selected $s_3 \in [1, \max(\{N, M\}) - (s_2 - s_1)]$. Finally, the sub-sequence with the indices $\in [s_1, s_2]$ from the smaller sequence is exchanged with the sub-sequence with indices $\in [s_3, s_3 + (s_2 - s)1]$ from the larger sequence, accounting for changes to bond connectivity as needed.
  - *Deletion*: For a given sequence of length N, the length of a sub-sequence for deletion $l_{\text{del}} \in [0, N - 20]$ is selected with uniform random probability. Next, an index s is chosen from the set $[0, N - l_{\text{del}}]$. Then, the amino acids with indices in the sequence $\in [s, s + l_{\text{del}} - 1]$ are removed from the chain. If necessary, bonds are established between the amino acids with indices of $s - 1$ and $s + l_{\text{del}}$.
  - *Growth*: For a given sequence, the length of a sub-sequence for addition $l_{\text{gro}} \in [0, 50 - N]$ is selected with uniform random probability. Next, a random position s in the sequence is selected with uniform probability. Then, the sub-sequence with indices $\in [1, l_{\text{gro}}]$ is replicated and inserted at s. As necessary, the bond between amino acids at s and $s + 1$ is eliminated, the amino acid at s is bonded to the first amino acid in the replicated sequence, and the amino acid formerly at $s+1$ is bonded the last amino acid in the replicated sequence.
  - *Mutation*: For a given sequence of length N, an index $s \in [1, N]$ is selected with uniform random probability. Then, the amino acid at position s is exchanged for another amino acid, which is selected from the pool of twenty amino acids with uniform random probability.

  The probabilities for crossover, deletion, growth, and mutation are 0.5, 0.2, 0.5, and 0.5, respectively. The moves are executed independently in the order of crossover, deletion, growth, and mutation. This procedure generates two new "child" sequences.

- *Step 3*: Repeat *Step 2* until $\lfloor 0.7n \rceil$ new sequences have been produced. These newly generated child sequences are combined with the top 30% of the parent sequences from the prior generation to comprise a new set of n parent sequences, which represent a new generation.

- *Step 4*: The fitness function is evaluated over the new generation of prospective parent sequences. The sequences are ranked by fitness.

- *Step 5*: Repeat starting from Step 2 until 100 generations have been produced.

- *Step 6:* The sequence with the best fitness at the conclusion of all generations is identified and proposed as a candidate for simulation as part of the active learning iteration.

Steps 1-6 above results in one candidate sequence. To facilitate convergence towards an optimal sequence, *Steps 1-6* are executed 96 times in parallel (see Fig. S11) without communication. The resulting 96 sequences, which differ by the stochastic nature of the genetic algorithm, are ranked by their fitness, and the one with the best overall fitness is marked for characterization by simulation. This overall workflow is then executed again (going back to *Step 1*) 95 additional times to obtain a total of 96 distinct sequences for characterization by simulation. Note that the fitness function changes based on the active learning iteration and the prior history of proposed sequences (see Eqn. 7 of the main text). Although a genetic algorithm was used for this task, any reasonable optimization algorithm should be able to achieve similar results. In addition, different move probabilities or move sets may facilitate better convergence but were not explored here.

To characterize the stochasticity of the sequences produced by the genetic algorithm (GA), we perform five replicate rounds of GA to generate alternative sequences for the final "exploitation" step of our active learning approach (see Figure 2c in the main text). The predicted B_2 and D values of these polypeptides are shown in Fig. S12, and representative sequences are listed in Tables S2–S5. In Fig. S12, we consider four distinct regions along the Pareto front as examples for detailed comparisons. Within each region, the sequences generated by different GA replicates are predicted to have similar B_2 and D values and thus are predicted to lie in close proximity to the Pareto front. However, as shown in Tables S2–S5, the sequences generated by different GA replicates differ substantially from one another due to the stochastic nature of the GA optimization approach. Nevertheless, in this exercise, we do not identify any sequences that notably improve upon our previously identified Pareto front. This suggests that our stochastic optimization has converged to a representative boundary in terms of the thermodynamics-dynamics tradeoff, although precise sequences would likely differ were the study to be conducted again.

1.5 Determination of Counterfactuals

The task of determining a counterfactual for a sequence requires identifying another sequence that possesses overall similar characteristics but exhibits a different classification. In the context of this study, the classifications correspond to *Pareto-optimal* versus *near-Pareto-optimal*. To treat our two objective properties (D and B_2) on equal footing, we consider the classification of near-Pareto-optimal to be based on a distance determined according to standard-normalized variables $\tilde{D}$ and $\tilde{B}_2$. Each sequence i can then be represented as a coordinate in the standard-normalized B_2–D plane as $\vec{z}^{(i)} = (\tilde{B}_2^{(i)}, \tilde{D}^{(i)})^\intercal$. To account for statistical uncertainties associated with the quantities obtained from simulations, we associated near-Pareto-optimal with distances $d \in [0.15, 0.3]$, which are measured from a given coordinate to a piecewise-linear approximation to the Pareto front. In particular, we first calculate three sets of 35 distances ($\{d_{v,i}\}$, $\{d_{1,i}\}$ and $\{d_{2,i}\}$) associated with our 35 Pareto-optimal sequences with coordinates $\vec{z}^{(\mathrm{P}_1)}, \ldots, \vec{z}^{(\mathrm{P}_{35})}$ as below:

$$d_{v,i} = \frac{\left(\vec{z}^{(k)} - \vec{z}^{(\mathrm{P}_i)}\right) \times \left(\vec{z}^{(\mathrm{P}_i)} - \vec{z}^{(\mathrm{P}_{i+1})}\right)}{\left|\left(\vec{z}^{(\mathrm{P}_i)} - \vec{z}^{(\mathrm{P}_{i+1})}\right|}, \tag{5}$$

$$d_{1,i} = |\vec{z}^{(k)} - \vec{z}^{(\mathrm{P}_i)}|, \tag{6}$$

and

$$d_{2,i} = |\vec{z}^{(k)} - \vec{z}^{(\mathrm{P}_{i+1})}|, \tag{7}$$

where $d_{v,i}$ is the distance between the point $\vec{z}^{(k)}$ to the piecewise lines determined by two adjacent Pareto-optimal points $\vec{z}^{(\mathrm{P}_i)}$ and $\vec{z}^{(\mathrm{P}_{i+1})}$, $d_{1,i}$ is the distance between $\vec{z}^{(k)}$ and $\vec{z}^{(\mathrm{P}_i)}$, and $d_{2,i}$ is the distance between $\vec{z}^{(k)}$ and $\vec{z}^{(\mathrm{P}_{i+1})}$; for the above $i \in [1, 34]$.

A series of distances $d_k^{(i)}$ for $\vec{z}^{(k)}$ are then determined as

$$d_k^{(i)} = \begin{cases} d_{v,i} & \text{if } \cos\theta_{1,i} > 0, \text{ and } \cos\theta_{2,i} < 0 \\ d_{1,i} & \text{if } \cos\theta_{1,i} < 0, \text{ and } \cos\theta_{2,i} < 0 \\ d_{2,i} & \text{if } \cos\theta_{1,i} > 0, \text{ and } \cos\theta_{2,i} > 0, \end{cases} \tag{8}$$

where $\theta_{1,i}$ is the angle between $(\vec{z}^{(k)} - \vec{z}^{(\mathrm{P}_{i+1})})$ and $(\vec{z}^{(\mathrm{P}_i)} - \vec{z}^{(\mathrm{P}_{i+1})})$, and $\theta_{2,i}$ is the angle between $(\vec{z}^{(k)} - \vec{z}^{(\mathrm{P}_i)})$ and $(\vec{z}^{(\mathrm{P}_i)} - \vec{z}^{(\mathrm{P}_{i+1})})$:

$$\cos\theta_{1,i} = \frac{(\vec{z}^{(k)} - \vec{z}^{(\mathrm{P}_{i+1})}) \cdot (\vec{z}^{(\mathrm{P}_i)} - \vec{z}^{(\mathrm{P}_{i+1})})}{|(\vec{z}^{(k)} - \vec{z}^{(\mathrm{P}_{i+1})})||(\vec{z}^{(\mathrm{P}_i)} - \vec{z}^{(\mathrm{P}_{i+1})})|}, \tag{9}$$

$$\cos\theta_{1,i} = \frac{(\vec{z}^{(k)} - \vec{z}^{(\mathrm{P}_i)}) \cdot (\vec{z}^{(\mathrm{P}_i)} - \vec{z}^{(\mathrm{P}_{i+1})})}{|(\vec{z}^{(k)} - \vec{z}^{(\mathrm{P}_i)})||(\vec{z}^{(\mathrm{P}_i)} - \vec{z}^{(\mathrm{P}_{i+1})})|}. \tag{10}$$

Finally, the shortest distance of $z^{(k)}$ to the Pareto front is set as

$$d_k = \min(\{d_k^{(i)}\}). \tag{11}$$

2 Supplementary Figures and Tables

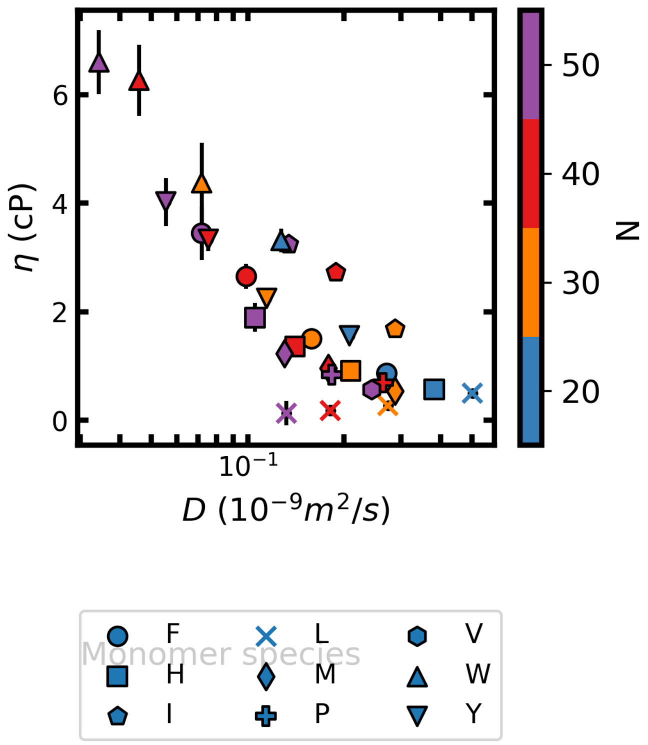

Figure S1: **Relationship of viscosity and self-diffusivity in the condensed-phase of homomeric polypeptides.** The viscosity, η, is anticorrelated with self-diffusivity, D, although the former possesses larger statistical uncertainties. The Pearson correlation coefficient between η and $log(D)$ is -0.91. For all systems, quantities are measured in the canonical ensemble at the estimated coexistence density of the condensed phase as determined by the approximate EOS method (see main manuscript). The viscosity is determined using the Green–Kubo formalism (85). Error bars represent the standard error of the mean.

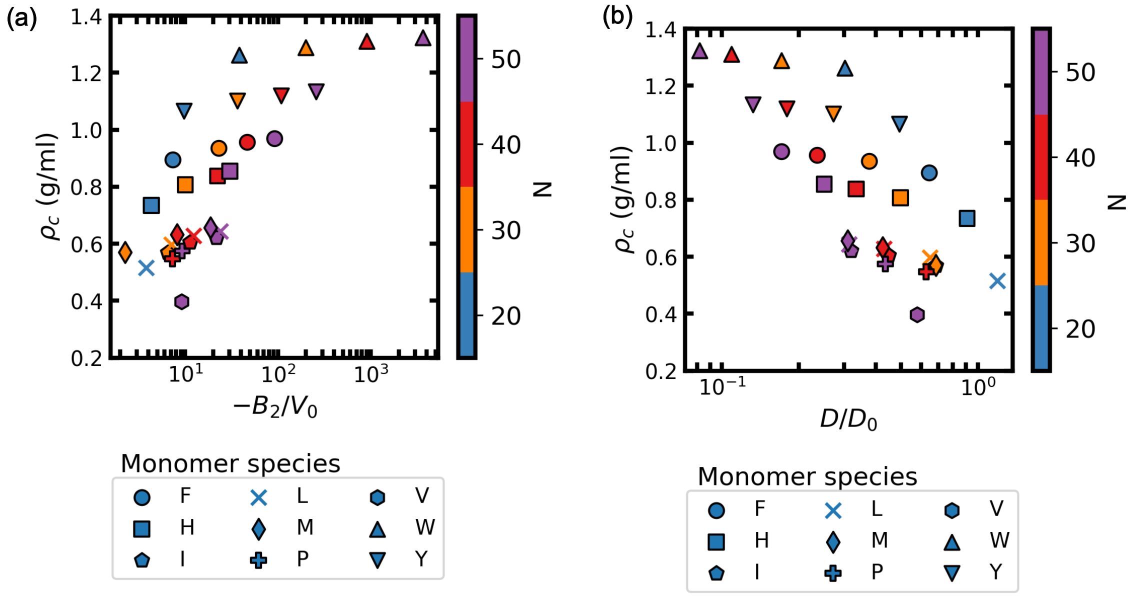

Figure S2: **Correlation of condensed-phase densities with target properties in phase-separating homomeric polypeptides.** Correlation plots for condensed-phase density, ρ_c with **(a)** the dimensionless second-virial coefficient, $-B_2/V_0$, and **(b)** the dimensionless self-diffusion coefficient in the condensed-phase, D/D_0. The Pearson correlation coefficients between ρ_c and $log(-B_2/V_0)$, and between ρ_{textc} and $log(D/D_0)$ are 0.80 and -0.77, respectively. Standard errors are comparable to symbol sizes.

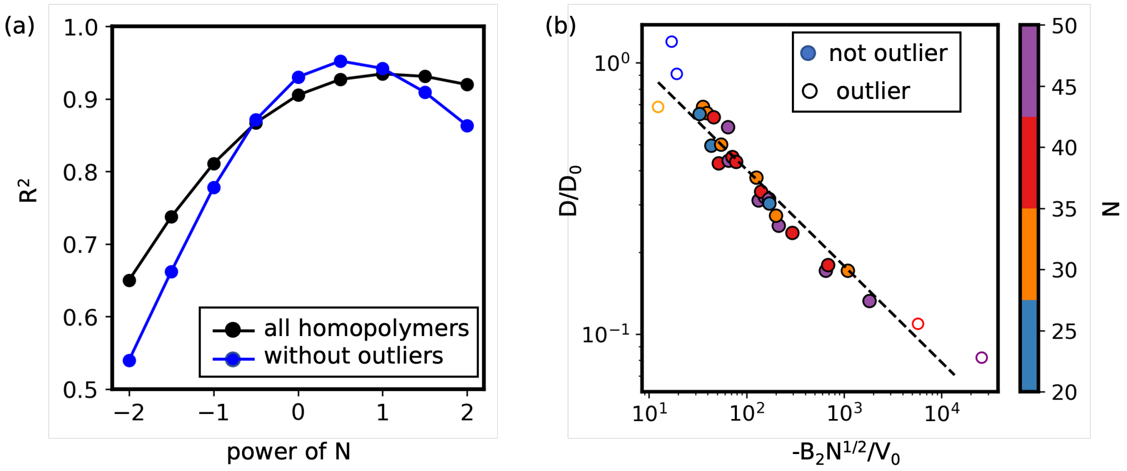

Figure S3: **Examination of thermodynamics-dynamics tradeoff for homomeric polypeptides. (a)** Coefficient of determination for correlation between dynamics via diffusion coefficient ($\log[D/D_0]$) versus a measure of thermodynamic attraction via $\log[-B_2 N^\nu/V_0]$ where B_2 is the second virial coefficient, N is the sequence length, and ν is a power-law scaling exponent. The highest correlation is for $\nu = 1/2$ or $\nu \approx 1$. **(b)** Illustration of data collapse with $\nu = 1/2$. In both panels, data points are considered "outliers" if $-B_2 > 10^{6.5}$ or $-B_2 < 10^{4.5}$.

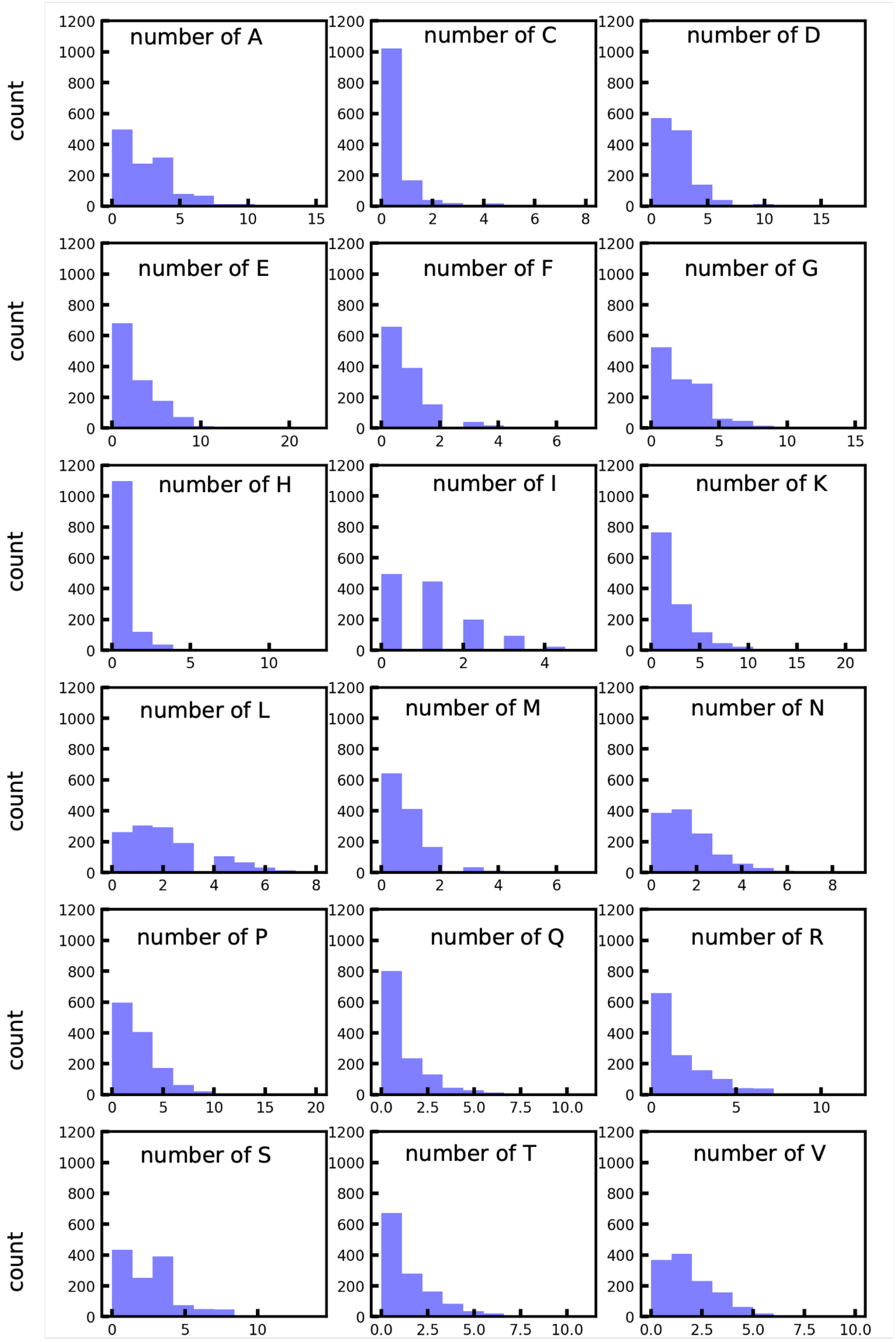

Figure S4: **Distribution of values for features among sequences selected from the DisProt database.** Panels show the number of each amino-acid type (A, C, D, E, F, G, H, I, K, L, M, N, P, Q, R, S, T, V, W, or Y) and the ten sequence characteristics described in SI Sec. 1.1. Sequences possess lengths of $20 \leq N \leq 50$.

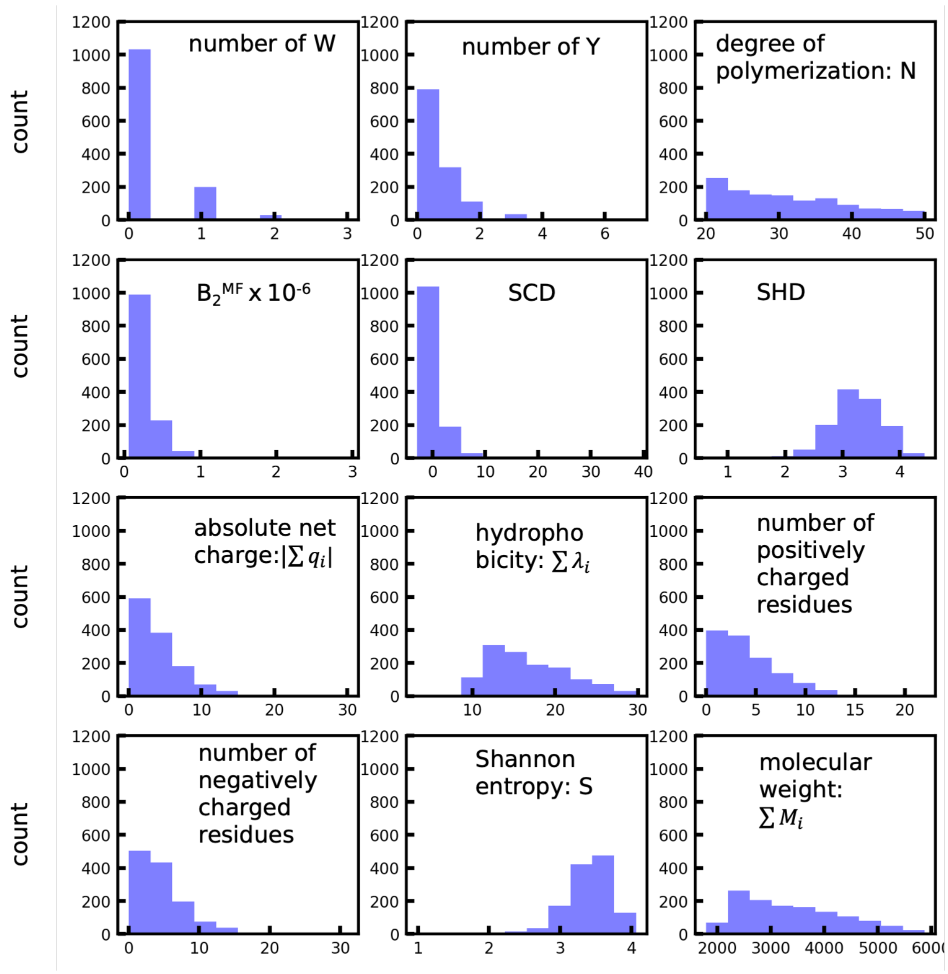

Figure S4 (Continued): **Distribution of values for features among sequences selected from the DisProt database.** Panels show the number of each amino-acid type (A, C, D, E, F, G, H, I, K, L, M, N, P, Q, R, S, T, V, W, or Y) and the ten sequence characteristics described in SI Sec. 1.1. Sequences possess lengths of $20 \leq N \leq 50$.

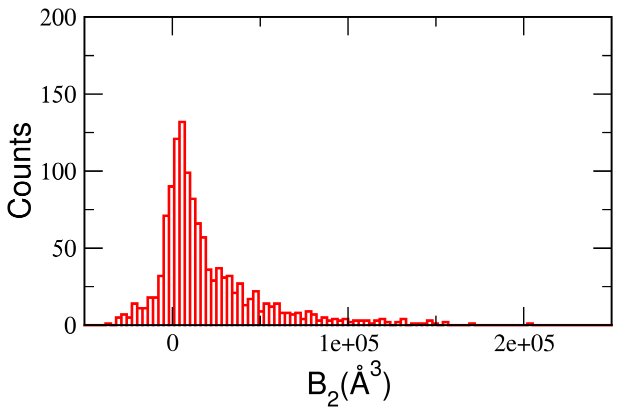

Figure S5: **Distribution of calculated second virial coefficients among sequences selected from the DisProt database.** Sequences possess lengths of $20 \leq N \leq 50$. The majority of sequences exhibit $B_2 > 0$.

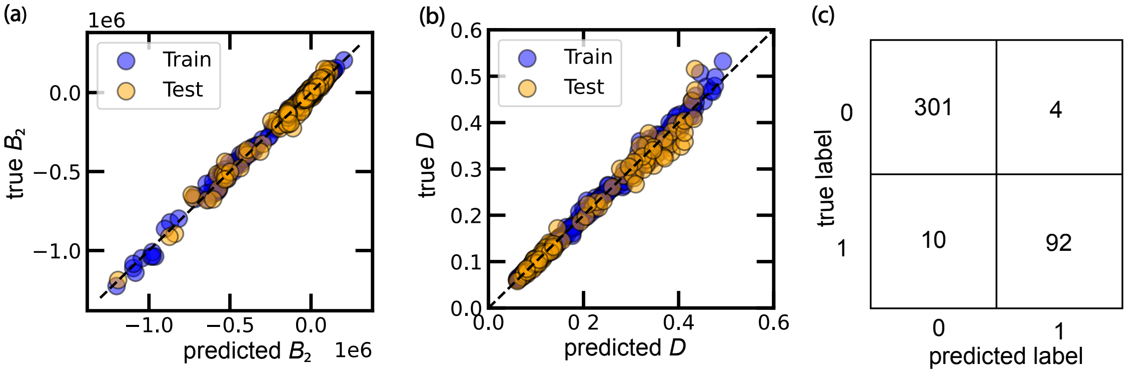

Figure S6: **Illustration of the quality of machine-learning models.** **a** Parity plot for the Gaussian process regression model that predicts the second-virial coefficient, B_2. **(b)** Parity plot for the Gaussian process regression model that predicts the condensed-phase self-diffusion coefficient, D . **(c)** Confusion matrix (test set) for classification of phase-separation behavior. A value of '0' indicates no phase separation and '1' indicates phase separation. The squares on the diagonal report the number of true classifications while the off-diagonal squares report the number of misclassifications. In all panels, the train-test split is 80% for training and 20% for testing with random selection. In **(a)** and **(c)**, all 2034 heteromeric sequences are included. In **(b)**, only the subset of the 2034 heteromeric sequences for which condensed-phase densities could be extracted were included; this includes 508 sequences.

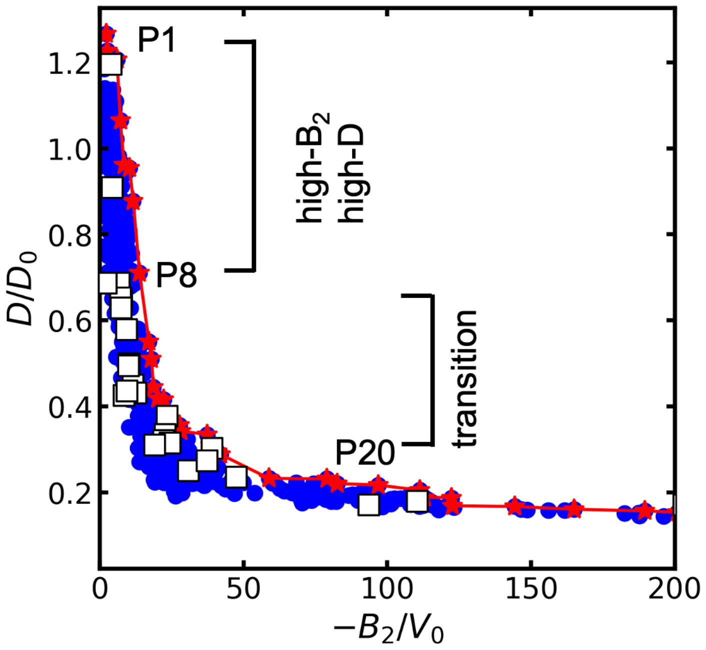

Figure S7: **The relationship between the dimensionless diffusivity, D/D_0, and the dimensionless second virial coefficient, $-B_2/V_0$, of all designed IDP sequences that undergo phase separation.** The plot features the same data as Fig. 2c of the main text but on linear scale and with all sequences indicated by markers. Red stars, blue circles, and white squares correspond to Pareto-optimal sequences, non-Pareto-optimal sequences, and homomeric polypeptides, respectively. The Pareto-optimal sequences P1, P8, and P20 are identified to outline the rough division of the high-B_2/high-D regime, transition regime, and low-B_2/low-D regime discussed in the main text.

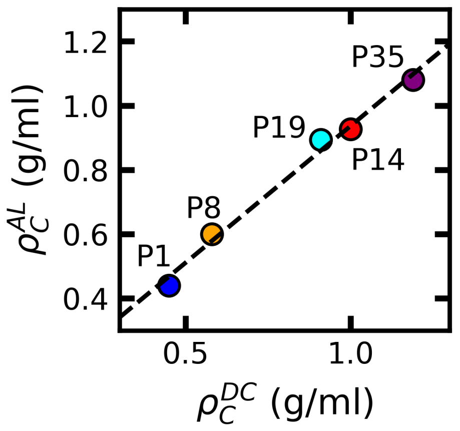

Figure S8: **Correlation of condensed-phase densities estimated within the active-learning (AL) framework or via direct-coexistence (DC) simulations.** Within the AL framework, the equation-of-state (EOS) method is used to extract estimates for condensate density that can be used for evaluation of dynamical properties. The DC simulations correspond to those shown in Figure 3a of the main text. The dashed line shows a linear fit to the data with a coefficient of determination of $R^2 = 0.99$ and a slope of 0.85, suggesting high correlation with consistent underestimates provided by the EOS method.

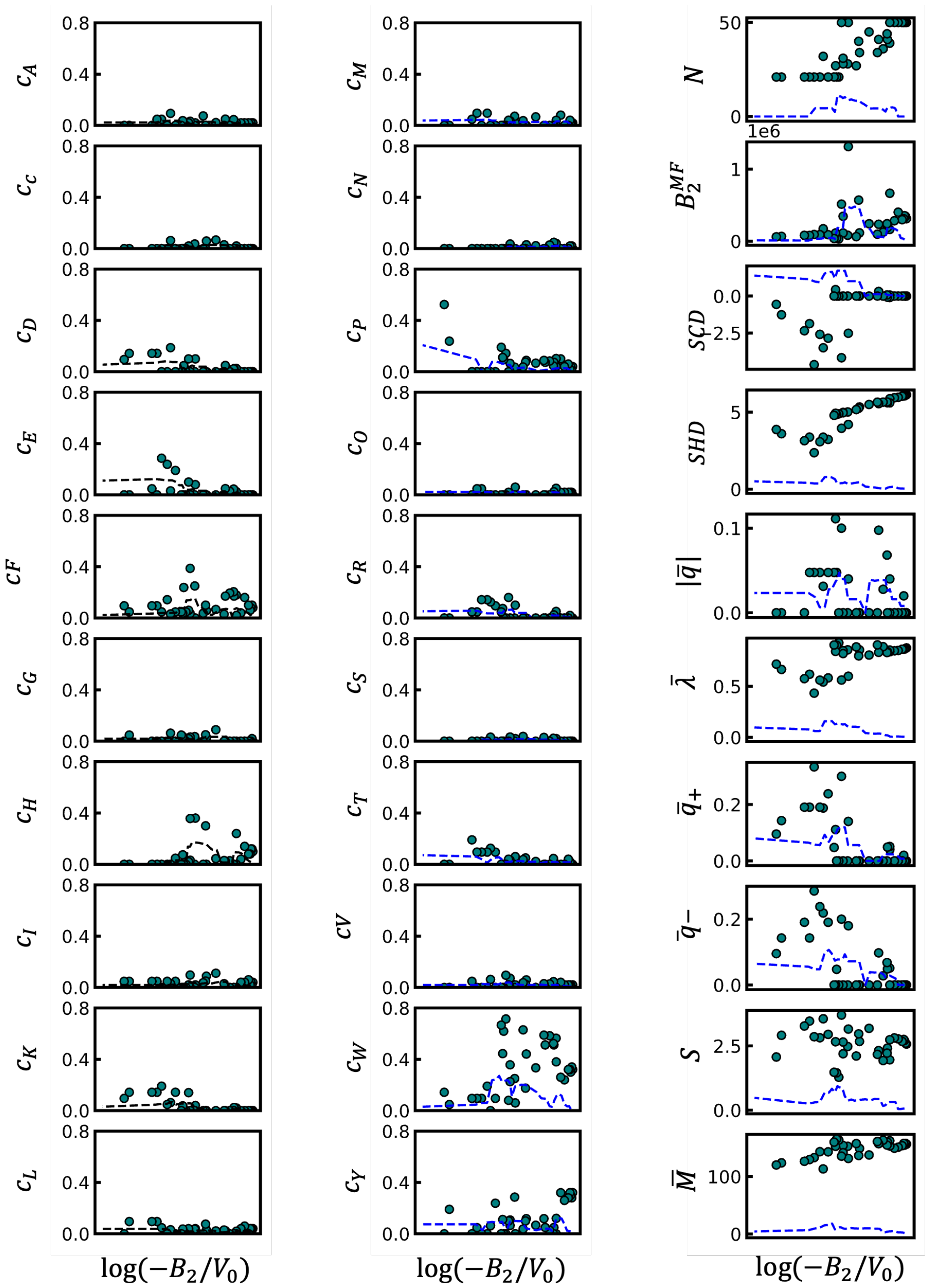

Figure S9: **Variation of feature values across the Pareto front.** Each marker indicates the value of a specific feature for a Pareto-optimal sequence. The data are presented such that both the diffusion coefficient and $-B_2$ are decreased moving left-to-right. The dashed lines indicate a rolling standard deviation of the feature x (i.e., $\sqrt{\frac{1}{m}\sum_{i=1}^{m}(x_i - \bar{x})^2}$, where $\vec{x}$ is the feature vector, the bin size $m = 5$, and $\bar{x} \equiv \frac{1}{m}\sum_{i=1}^{m} x_i$) as measure of the extent of variability of a feature in particular regions of the Pareto front.

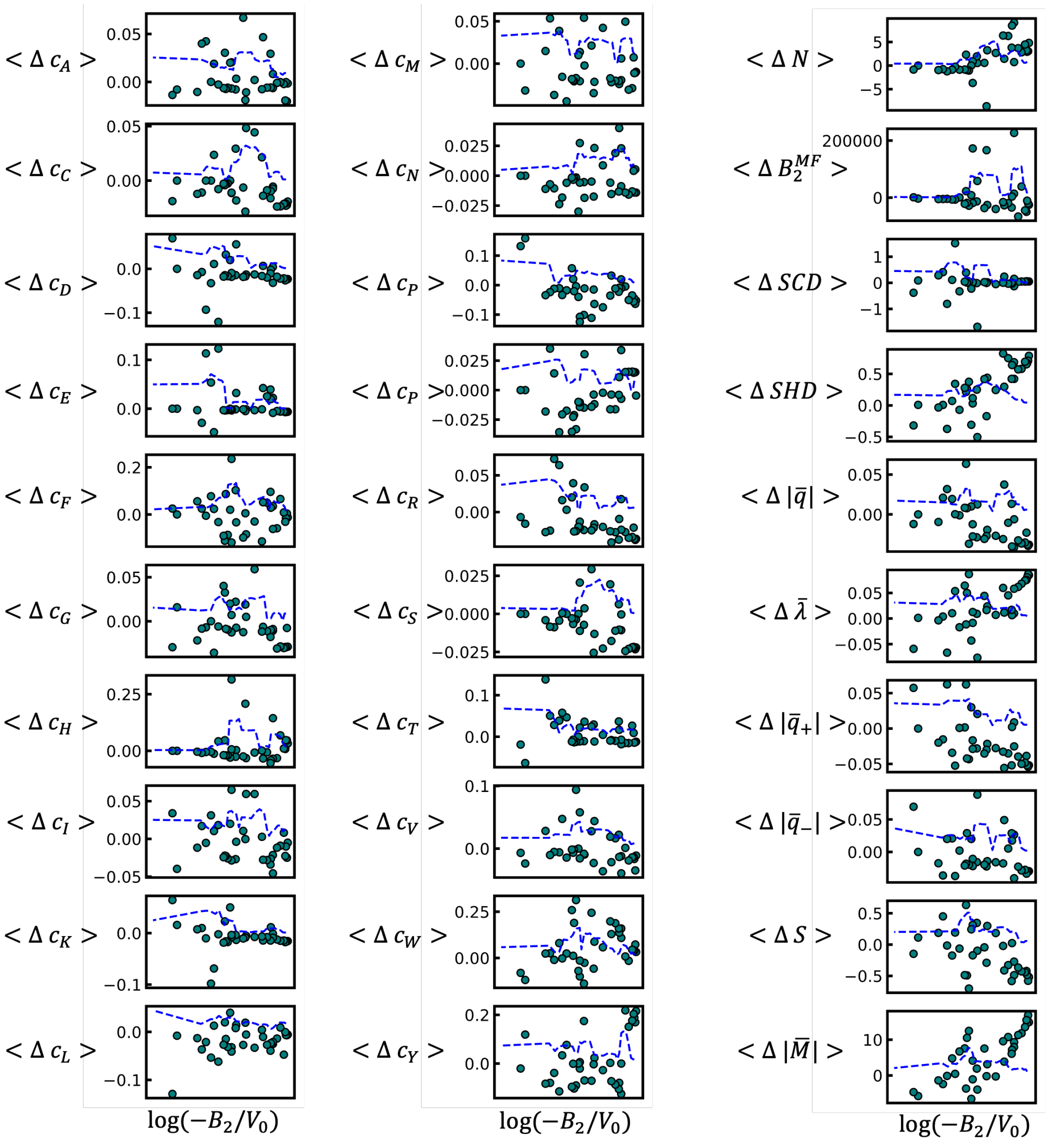

Figure S10: **Average differences in feature values between Pareto-optimal sequences and their counterfactuals.** Average feature differences determined via Eq. (2) of the main text. The dashed lines indicate a rolling standard deviation as a measure of the extent of variability of the feature differences in particular regions of the Pareto front (see SI Fig. S9).

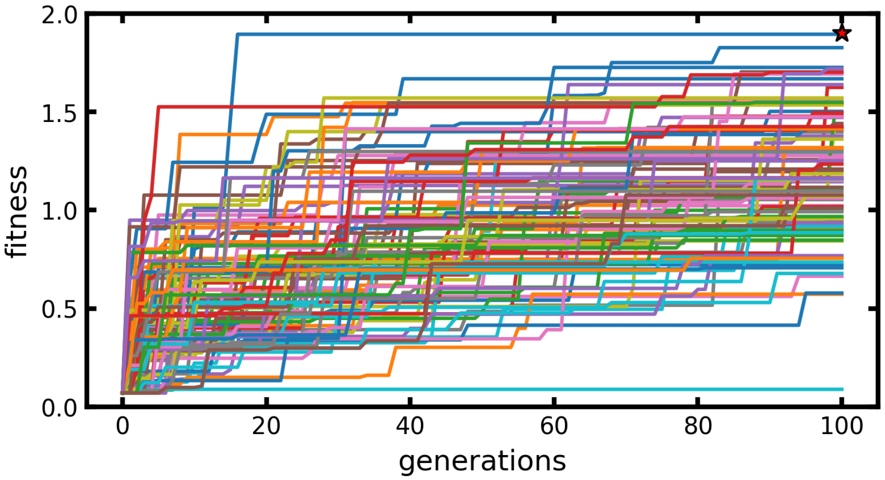

Figure S11: **Illustration of convergence toward an optimal sequence using parallel execution of the genetic algorithm.** Each solid line indicates the current highest fitness of a sequence produced over the course of 100 generations for a single execution of the genetic algorithm (*Steps 1- 6* in Sec. 1.4). There are 96 lines corresponding to 96 independent executions with the same set of possible parent sequences. The different executions lead to a range of sequences with different fitness scores. The single best sequence (red star) is proposed for characterization by molecular dynamics simulation. A given active learning iteration produces 96 sequences in this manner.

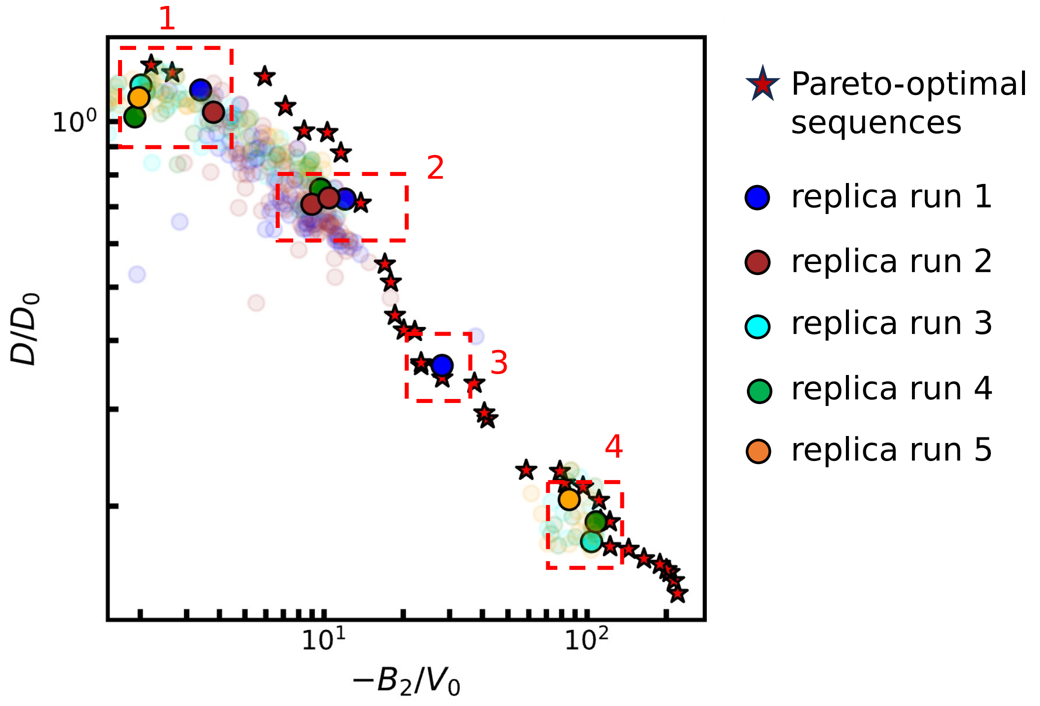

Figure S12: **Comparison of optimized sequences produced during replicate runs of the genetic algorithm.** The machine-learning predicted values of B_2 and D for sequences generated by five replicate runs of the genetic algorithm (GA) in "exploitation" mode (see Figure 2c in the main text). The final Pareto front (red stars; cross referenced in Figure 2b in the main text) is shown for comparison. We compare representative sequences (opaque solid circles), each generated by a different GA replicate, from four distinct regions, 1–4, along the Pareto front to understand the stochasticity of the genetic algorithm. These replicate sequences are listed in Tables S2–S5. Sequences not listed in the tables are shown as partially transparent circles for clarity.

Table S1: Pareto-optimal sequences

P35	YWWWWWYWPYFHYWWWWYWFMWWHIFYWLWVHYYLYWWYFYYYHPYIWYHYA
P34	YWWWWWYWPYFHYWWWWYWFMWWHIFYWHWWHYYLYHWYFYYWQNYGWYHLF
P33	YWWWWWYWPYFHYWWWWYWFMWWHIFYWHWVHYYLYHWYFYYYQPYIWYHLA
P32	YWWWWWYWPYFHYWWWWYWFTWWHIYYWHWVYYYLYWWYFYYYQMNMWYHLR
P31	YWWWWWYWPYFFYWWWWYWFPWWTIFYWHWVHYTYYHWYFWYYQPYIIYHWA
P30	WFWWYFLPYFHYFWWFWFIWWHHFYWHWVHYYLYHWYFYYYQPYIWYHLA
P29	YWWWWWYWPYFPYWWWWYWPMWYMIFYWHYVPYYLYYWYMYVYQPYHWIMLA
P28	MHWYWHWNHWWNWYHHWWYWKYSHPWWFLWAPRWHWWHHHMYYWWHWWCH
P27	RQFWFPEDRFQWFFWWWWWWWWFWPWWWWWWWWFWPWWWWP
P26	DKRQFFEWWWTWNFWYFFYNFWWWWWWWWFWPWWWWPWWWWP
P25	AEEDTVWAFFWVFWWWSWFWFWPWTFWFFWWWWWWWWFWPWWWWW
P24	VHFWFYWWWNWWWPWPEWWFWFWFFWWWWWWWWFWWP
P23	DFLEFDFYWEFWWHWYAWFAPWWWWFWWWWWWWWFWPWWWWP
P22	PWWFWWWLWWWWWSWPWWWHWYCWYWPVWIYWWY
P21	GFWPYGPIWWWWWCIWIIWYLFGFWMWHWANIMHCGWWWWYPMCT
P20	WIWNWGCLVIFWWPWPWWWFWYCWYWPVWIYWWY
P19	GPHWVHWPHHPYHHWWWHHLTLFYWHIYFHGWFFIHTHYL
P18	IPWWWWFWWWWWATWWWWYMWWSWWVA
P17	HEWDDEDWTQHHHHHAHEHHHHRTIEWQTDHDVLRHHHKQHMSRKLHRHR
P16	WFWWYYLWYYMWWYMFGFFYYPFFWYFC
P15	MHWYWHWNHWWHWYHHWWHFVWVHHYWH
P14	FWWFWPWIGFFFWIFYSFFFTWIFPHWFFYA
P13	YEDDDFERDEDKWYGTFEFLWKHMRQYYKCREKARIWKYVRRWKLKMRRT
P12	PWWFWWWPWWWWWVVWPWWWW
P11	DFFWFFWWWWWWWWFWPWWWWP
P10	RAYWWHGWPWWWPLPWYWWFHWRWWYK
P9	PPWPGPWWWWRWWWFWWWWWW
P8	EEEEWIHYTTYWYYFYKKRRK
P7	TKRGRTARKTGALCRTYFACYSDDDVDVDIDE
P6	RRRKMTFWWWTFWIEEFMEEE
P5	RRRKKKFKWQTLWAEETEEEE
P4	DDDMQAFLTFTWWYLIMRKKK
P3	DDEMDTTLTFTWWFLIVRKKK
P2	KKKLPYLPIGYFWYDPPDYPD
P1	KKPPWPWPFPIPDPWFPPPPD

Table S2: Representative sequences in the region 1 of Fig. S12 generated by five GA replicates. The Pareto-optimal sequence P1 study is shown for comparison.

**Replicate**	**Sequence**
P1	KKPPWPWPFPIPDPWFPPPPD
1	IGLRAQTWPPMWQQWILPQPD
2	DWWEDLQYPQPFRKQMWRWEK
3	KKPTWPWPGPIPFPWFPSPPF
4	RRRCKKMDYTHMPDLITDTED
5	YDNTFRQCTPGMWFIVCLWMM

Table S3: Representative sequences in the region 2 of Fig. S12 generated by five GA replicates. The Pareto-optimal sequence P8 is shown for comparison.

**Replicate**	**Sequence**
P8	EEEEWIHYTTYWYYFYKKRRK
1	TWRPRTARKTFAMPYTYWYCYSDDDVDEDNL
2	DDEGDCGPCDDCMPFKPEFVLLTFTWWFLIVRKKK
2	DDDDIPQLWDSRTFKILKFLIWFLIVRKKK
4	RRRKKKLRSKKKFCWQTEWAEETEEEE

Table S4: Representative sequences in the region 3 of Fig. S12 generated by five GA replicates. The Pareto-optimal sequences P14 and P15 are shown for comparison.

Replicate	Sequence
P14	FWWFWPWIGFFFWIFYSFFFTWIFPHWFFYA
P15	MHWYWHWNHWWHWYHHWWHFVWVHHYWH
1	DDNTWDWNHMVPEHHDDVIFHWNHWWHWYHHWWHWWHFVWVHHYWH

Table S5: Representative sequences in the region 4 of Fig. S12 generated by five GA replicates. The Pareto-optimal sequences P21–P24 are shown for comparison.

Replicate	Sequence
P21	GFWPYGPIWWWWWCIWIIWYLFGFWMWHWANIMHCGWWWWYPMCT
P22	PWWFWWWLWWWWWSWPWWWHWYCWYWPVWIYWWY
P23	DFLEFDFYWEFWWHWYAWFAPWWWWFWWWWWWWFWPWWWWP
P24	VHFWFYWWWNWWWPWPEWWFWFWFFWWWWWWWWFWWP
3	QRWFPWWKESTWWESHWWWFWYCRWWFWWWLWWWWWYWPVWIYWWY
4	WWRHWYWPPHGQYQWDLQPWHWLPWVYWWHWYWWWFWLYIYPFYWPHYW
5	QAWRWHWIRWRIWWHWWAYHQHWWWWRWWWFWWWWWWWWW

Table S6: Counterfactual sequences for Pareto-optimal sequences

$\mathcal{C}_{1,1}$	PWLYWWWPFPWPPIFPWPPPP	$\mathcal{C}_{1,2}$	FFCWPWPLPLPPPLPPFFVWP
$\mathcal{C}_{1,3}$	DLDLLLPYAWPWLLWYLFLKK	$\mathcal{C}_{1,4}$	PWPLWAWLPLPTGPPPWCPLP
$\mathcal{C}_{1,5}$	LLPWFTPPPWWWLWWPPWGPP	$\mathcal{C}_{1,6}$	PPIPPPPWWDWPWPWPWKPPR
$\mathcal{C}_{1,7}$	KKPPGPPPGPWWWWFPCTGFWFPPPPD		
$\mathcal{C}_{2,1}$	DDDPFLTYYWWFYPTFGLKKK	$\mathcal{C}_{2,2}$	DDDLYLWFYWWPLPPWPRKPK
$\mathcal{C}_{2,3}$	DDGDWWWIWGTWTPPKPKKWW	$\mathcal{C}_{2,4}$	DDDIWIIWVYIIIILKIRKIT
$\mathcal{C}_{2,5}$	KKKVWMWLMMLDLWLLMDLDV	$\mathcal{C}_{2,6}$	DDDGTAFWTFTWYYIWLKKKY
$\mathcal{C}_{3,1}$	TWEEEEYDWYTQWMNKKKKYK	$\mathcal{C}_{3,2}$	LDDDDWGLLCYLYFWCTRKKK
$\mathcal{C}_{3,3}$	TRKKKTTPWTRWTFTDEDDDT	$\mathcal{C}_{3,4}$	EDEVDYYTVQTMWWTYYMRKKK
$\mathcal{C}_{3,5}$	GDDDDYYPDPYVPYKFRKKRY	$\mathcal{C}_{3,6}$	DGDDDDLYVPYCWLYPRKKRK
$\mathcal{C}_{3,7}$	RKKKIPPWIPGYWADYWGDDD	$\mathcal{C}_{3,8}$	KRKKKTLNQWFLMLWDIDDEE
$\mathcal{C}_{3,9}$	KKKMKLLGYYYYWMLIWDDDD	$\mathcal{C}_{3,10}$	DEDEIIWIILLFYYLLFRKKK
$\mathcal{C}_{3,11}$	EELEEEWWLLFLMWRLQKKRK	$\mathcal{C}_{3,12}$	KKRVKLTLLTWTLFHLDDDDL
$\mathcal{C}_{3,13}$	AKIIRINIKRKIWRIMLGWGEDEENDYD	$\mathcal{C}_{3,14}$	WDDDDILLLNVLYWPKRKKKL
$\mathcal{C}_{3,15}$	KKKRYLLIQWLILQWIEQDED	$\mathcal{C}_{3,16}$	DDDPDYPIYIGMIPYIRRCRR
$\mathcal{C}_{3,17}$	DDDDDYGLYVILPIFGRKRKK	$\mathcal{C}_{3,18}$	MLLRKRRLYFSMWIDDTDYDS
$\mathcal{C}_{3,19}$	DDDMDTTLTFTCYYFYRKRRK	$\mathcal{C}_{3,20}$	DDDDLMDTQLTFTWWFLIHRKKK
$\mathcal{C}_{3,21}$	DDDDMMDTVPPAYAYFFWRELIVRKKK	$\mathcal{C}_{3,22}$	RRFKKKFIWQVCWLPAYLDEFDE
$\mathcal{C}_{3,23}$	LLDEDAIEIWMLDFNLWMLIRRKKK		
$\mathcal{C}_{4,1}$	YDDDMEWTYFYMWTTKRKKYK	$\mathcal{C}_{4,2}$	LDDDDWGLLCYLYFWCTRKKK
$\mathcal{C}_{4,3}$	EDDLDYWFLYFFFFFRKMKKL	$\mathcal{C}_{4,4}$	DDDACPYYWIWYYYPWPKKKK
$\mathcal{C}_{4,5}$	EDEVDYYTVQTMWWTYYMRKKK	$\mathcal{C}_{4,6}$	KKYFKRRPWFYFFFFDGPDDD
$\mathcal{C}_{4,7}$	KKKMKLLGYYYYWMLIWDDDD	$\mathcal{C}_{4,8}$	DEDEIIWIILLFYYLLFRKKK
$\mathcal{C}_{4,9}$	DTDDYYDTYTYYYFLLRKKKK	$\mathcal{C}_{4,10}$	DDHEDNQHQNLTWYPMLFMRRTKMKKH
$\mathcal{C}_{4,11}$	YDDEDLWNYLYILLRLLRRRK	$\mathcal{C}_{4,12}$	KKKYKSWLLLLYLLILWDDWD
$\mathcal{C}_{4,13}$	DDDYLYYLDLYQLLLLRLRRR	$\mathcal{C}_{4,14}$	YDDDDMHLTFRWMFLIPRKKK
$\mathcal{C}_{4,15}$	MLLRKRRLYFSMWIDDTDYDS	$\mathcal{C}_{4,16}$	DDEMMLDAFWTLPFTWWYTRVRKKK
$\mathcal{C}_{4,17}$	RRFKKKFIWQVCWLPAYLDEFDE		
$\mathcal{C}_{5,1}$	KKKQRKKTKYKRKYKEEEPSDQGEPEDGDEDE	$\mathcal{C}_{5,2}$	TWEEEEYDWYTQWMNKKKKYK
$\mathcal{C}_{5,3}$	KRKKKKVLLMMWWHLLEDDDD	$\mathcal{C}_{5,4}$	TRKKKTTPWTRWTFTDEDDDT
$\mathcal{C}_{5,5}$	KKKKWWKKKFFMYWDEEFDDE	$\mathcal{C}_{5,6}$	KRKKKTLNQWFLMLWDIDDEE
$\mathcal{C}_{5,7}$	EELEEEWWLLFLMWRLQKKRK	$\mathcal{C}_{5,8}$	WLEEEEEETFMWFTFYKRRKR
$\mathcal{C}_{5,9}$	RRRKKQFKWQWSWEEEYAEEE		
$\mathcal{C}_{6,1}$	YDDDMEWTYFYMWTTKRKKYK	$\mathcal{C}_{6,2}$	TWEEEEYDWYTQWMNKKKKYK
$\mathcal{C}_{6,3}$	KYTKWYKRQYWLQYMYEYEEE	$\mathcal{C}_{6,4}$	EELEEEWWLLFLMWRLQKKRK
$\mathcal{C}_{6,5}$	WLEEEEEETFMWFTFYKRRKR	$\mathcal{C}_{6,6}$	KRKKKFWQWFQWMMEIWFEEE
$\mathcal{C}_{6,7}$	NRRKKNNYHNTRWEIYSEDDEMWETYMP	$\mathcal{C}_{6,8}$	EEEEWILYPVYLYFFFKRRRK
$\mathcal{C}_{7,1}$	RPKQKCRKGMYRPTNMFPKGPGKDEDDDDDED	$\mathcal{C}_{7,2}$	KGKALAKYKKQKKKIKFICATGDTDDPDDEDE
$\mathcal{C}_{7,3}$	KKRKARRRPLKSKLGCISEGNGPEDDDDEGTD	$\mathcal{C}_{7,4}$	KKRKKAARKCNLKQGSTPASQEEEDDCDGEDE
$\mathcal{C}_{7,5}$	KRAARKGRKKGLRCMERVSGNDGSDFDDDEEE	$\mathcal{C}_{7,6}$	KARKKRRARSCHKGANTEGPNDDADETCDEDD
$\mathcal{C}_{7,7}$	DEDTEEDGACESAEVSEQPGKGTRKVHKKKARKKR	$\mathcal{C}_{7,8}$	KRRGKQAQKYKTAPKGPGYIDDTSDDDQDDDE
$\mathcal{C}_{7,9}$	TKIGRMRRKTGACGRTGYACYTHDCDDVDHDD	$\mathcal{C}_{7,10}$	TMRGRTRRKTGYSGRTGYACTNDDDDDVDEML
$\mathcal{C}_{7,11}$	AKRGRIRRKTGAGIHTCRAIYEDDSADADEDE	$\mathcal{C}_{7,12}$	TPRGRTRRKTGALGRTGYACYADDDDDVDEDW
$\mathcal{C}_{8,1}$	YDDDMEWTYFYMWTTKRKKYK	$\mathcal{C}_{8,2}$	KKKKIFKKYYYMFHYWWYDEDIFE
$\mathcal{C}_{8,3}$	DDEWDIPFVYYWFNYKRKRRK	$\mathcal{C}_{8,4}$	ETEECDDFWRYDIMKMIYITKKRWKFMYR
$\mathcal{C}_{8,5}$	EDEVDYYTVQTMWWTYYMRKKK	$\mathcal{C}_{8,6}$	KKYFKRRPWFYFFFFDGPDDD
$\mathcal{C}_{8,7}$	DYDDDYMYLYYYRRLKWYKYK	$\mathcal{C}_{8,8}$	KYTKWYKRQYWLQYMYEYEEE
$\mathcal{C}_{8,9}$	DTDDYYDTYTYYYFLLRKKKK	$\mathcal{C}_{8,10}$	YDDEDLWNYLYILLRLLRRRK
$\mathcal{C}_{8,11}$	DDYDYWDYYLLMWLKRRKRKG	$\mathcal{C}_{8,12}$	KRKKKFWQWFQWMMEIWFEEE
$\mathcal{C}_{8,13}$	YDDDDMHLTFRWMFLIPRKKK	$\mathcal{C}_{8,14}$	DDDMDTTLTFTCYYFYRKRRK
$\mathcal{C}_{8,15}$	EEEEWILYPVYLYFFFKRRRK	$\mathcal{C}_{8,16}$	RRFKKKFIWQVCWLPAYLDEFDE

Table S6 continued:

$\mathcal{C}_{9,1}$	WPWWFHWWRRHWWYWYWWFFT	$\mathcal{C}_{9,2}$	WHHWYTWDWLWMYWWWWWFWD
$\mathcal{C}_{9,3}$	WFWFWPFPWPYPYWPWPWYPP	$\mathcal{C}_{9,4}$	WYWWWWHWFWLRRWWIEWWKW
$\mathcal{C}_{9,5}$	PPPWYPWWFMWWYFPPPPPPW	$\mathcal{C}_{9,6}$	WGWPIPWWWLWWWWWWAWWAW
$\mathcal{C}_{9,7}$	WWWIWWFHWHWWWFDFRWWWW	$\mathcal{C}_{9,8}$	YYYYWYYYMYLWYYYWSWYYY
$\mathcal{C}_{9,9}$	WWMYRYWWWWRYWHWFWPYFWWWRW	$\mathcal{C}_{9,10}$	FIWWPGGAPWFWWWFWWWWAGPPWGWLFF
$\mathcal{C}_{9,11}$	QQWWWWWQWQWWWWWMIIWWQ	$\mathcal{C}_{9,12}$	WYWYYIWWYYYIYRYYYWKYY
$\mathcal{C}_{9,13}$	WPFWWFFWPPFHFFFWWFPWF	$\mathcal{C}_{9,14}$	FWPGWRPYWWWWWPWWPPFWP
$\mathcal{C}_{9,15}$	WFFFRYRWYWFWWWWWDWWWW	$\mathcal{C}_{9,16}$	FFYFFMYFWFYFYFFFYWFFF
$\mathcal{C}_{9,17}$	WWWWWRQWNQWWMWWWRQWWW	$\mathcal{C}_{9,18}$	FWWFWWWFFFFKFFYFWWWWF
$\mathcal{C}_{9,19}$	PPWPPWWWWPWPWWMVWWIPW	$\mathcal{C}_{9,20}$	PWLYWWWPFPWPPIFPWPPPP
$\mathcal{C}_{9,21}$	RIFYFWWPWWHWWPWPFFRYPPFPYWAIW	$\mathcal{C}_{9,22}$	DFHWWWWYWYWWWWFFFWWDWSDI
$\mathcal{C}_{9,23}$	FWTWWPIIFFTWLWLTLPCWFWWP	$\mathcal{C}_{9,24}$	MQTFCWWFQWWWMPWWWPWFW
$\mathcal{C}_{9,25}$	EWWYMWMWQWQWWWWWFWKKK	$\mathcal{C}_{9,26}$	FFFFPHPWWFFFFPFFFWFWW
$\mathcal{C}_{9,27}$	LWPFWWPWPYWPWWPWFPYFP	$\mathcal{C}_{9,28}$	LWDDHWWLLIYLLLFLLRRKW
$\mathcal{C}_{9,29}$	PPIPPPPWWDWPWPWPWKPPR	$\mathcal{C}_{9,30}$	TWDDPWPYTWPYYWWPWPKKK
$\mathcal{C}_{9,31}$	WWQQQQQKQQWQQFYYQWQWW		
$\mathcal{C}_{10,1}$	WPWWFHWWRRHWWYWYWWFFT	$\mathcal{C}_{10,2}$	WHHWYTWDWLWMYWWWWWFWD
$\mathcal{C}_{10,3}$	WFWFWPFPWPYPYWPWPWYPP	$\mathcal{C}_{10,4}$	WYWWWWHWFWLRRWWIEWWKW
$\mathcal{C}_{10,5}$	WGWPIPWWWLWWWWWWAWWAW	$\mathcal{C}_{10,6}$	WWWIWWFHWHWWWFDFRWWWW
$\mathcal{C}_{10,7}$	YYYYWYYYMYLWYYYWSWYYY	$\mathcal{C}_{10,8}$	WWMYRYWWWWRYWHWFWPYFWWWRW
$\mathcal{C}_{10,9}$	QQWWWWWQWQWWWWWMIIWWQ	$\mathcal{C}_{10,10}$	WYWYYIWWYYYIYRYYYWKYY
$\mathcal{C}_{10,11}$	WPFWWFFWPPFHFFFWWFPWF	$\mathcal{C}_{10,12}$	FWPGWRPYWWWWWPWWPPFWP
$\mathcal{C}_{10,13}$	WFFFRYRWYWFWWWWWDWWWW	$\mathcal{C}_{10,14}$	FFYFFMYFWFYFYFFFYWFFF
$\mathcal{C}_{10,15}$	SCYRPPIFYFPWRRRPLWWVWWYWYTTGYIRWLWYHHAWWALSWPP	$\mathcal{C}_{10,16}$	WWWWWRQWNQWWMWWWRQWWW
$\mathcal{C}_{10,17}$	FWWFWWWFFFFKFFYFWWWWF	$\mathcal{C}_{10,18}$	PPWPPWWWWPWPWWMVWWIPW
$\mathcal{C}_{10,19}$	RIFYFWWPWWHWWPWPFFRYPPFPYWAIW	$\mathcal{C}_{10,20}$	DFHWWWWYWYWWWWFFFWWDWSDI
$\mathcal{C}_{10,21}$	MQTFCWWFQWWWMPWWWPWFW	$\mathcal{C}_{10,22}$	EWWYMWMWQWQWWWWWFWKKK
$\mathcal{C}_{10,23}$	FFFFPHPWWFFFFPFFFWFWW	$\mathcal{C}_{10,24}$	LWPFWWPWPYWPWWPWFPYFP
$\mathcal{C}_{10,25}$	KWVWNWHRYFHFHVYWLFYWYWRHWYWPWHFFDIWFWLYLLPFYWNCYW	$\mathcal{C}_{10,26}$	DQWLFVDRWVWWRLWKWWKSKWLWWWWWSWPWWWHWYCWYWPVWIYWWY
$\mathcal{C}_{11,1}$	WPWWFHWWRRHWWYWYWWFFT	$\mathcal{C}_{11,2}$	WHHWYTWDWLWMYWWWWWFWD
$\mathcal{C}_{11,3}$	WFWFWPFPWPYPYWPWPWYPP	$\mathcal{C}_{11,4}$	WYWWWWHWFWLRRWWIEWWKW
$\mathcal{C}_{11,5}$	PPPWYPWWFMWWYFPPPPPPW	$\mathcal{C}_{11,6}$	WGWPIPWWWLWWWWWWAWWAW
$\mathcal{C}_{11,7}$	WWWIWWFHWHWWWFDFRWWWW	$\mathcal{C}_{11,8}$	YYYYWYYYMYLWYYYWSWYYY
$\mathcal{C}_{11,9}$	WWMYRYWWWWRYWHWFWPYFWWWRW	$\mathcal{C}_{11,10}$	FIWWPGGAPWFWWWFWWWWAGPPWGWLFF
$\mathcal{C}_{11,11}$	QQWWWWWQWQWWWWWMIIWWQ	$\mathcal{C}_{11,12}$	WYWYYIWWYYYIYRYYYWKYY
$\mathcal{C}_{11,13}$	WPFWWFFWPPFHFFFWWFPWF	$\mathcal{C}_{11,14}$	FWPGWRPYWWWWWPWWPPFWP
$\mathcal{C}_{11,15}$	WFFFRYRWYWFWWWWWDWWWW	$\mathcal{C}_{11,16}$	FFYFFMYFWFYFYFFFYWFFF
$\mathcal{C}_{11,17}$	WWWWWRQWNQWWMWWWRQWWW	$\mathcal{C}_{11,18}$	FWWFWWWFFFFKFFYFWWWWF
$\mathcal{C}_{11,19}$	PPWPPWWWWPWPWWMVWWIPW	$\mathcal{C}_{11,20}$	RIFYFWWPWWHWWPWPFFRYPPFPYWAIW
$\mathcal{C}_{11,21}$	DFHWWWWYWYWWWWFFFWWDWSDI	$\mathcal{C}_{11,22}$	FWTWWPIIFFTWLWLTLPCWFWWP
$\mathcal{C}_{11,23}$	MQTFCWWFQWWWMPWWWPWFW	$\mathcal{C}_{11,24}$	EWWYMWMWQWQWWWWWFWKKK
$\mathcal{C}_{11,25}$	FFFFPHPWWFFFFPFFFWFWW	$\mathcal{C}_{11,26}$	LWPFWWPWPYWPWWPWFPYFP
$\mathcal{C}_{11,27}$	TWDDPWPYTWPYYWWPWPKKK		
$\mathcal{C}_{12,1}$	WPWWFHWWRRHWWYWYWWFFT	$\mathcal{C}_{12,2}$	WHHWYTWDWLWMYWWWWWFWD
$\mathcal{C}_{12,3}$	WFWFWPFPWPYPYWPWPWYPP	$\mathcal{C}_{12,4}$	WYWWWWHWFWLRRWWIEWWKW
$\mathcal{C}_{12,5}$	PPPWYPWWFMWWYFPPPPPPW	$\mathcal{C}_{12,6}$	WGWPIPWWWLWWWWWWAWWAW
$\mathcal{C}_{12,7}$	WWWIWWFHWHWWWFDFRWWWW	$\mathcal{C}_{12,8}$	YYYYWYYYMYLWYYYWSWYYY
$\mathcal{C}_{12,9}$	WWMYRYWWWWRYWHWFWPYFWWWRW	$\mathcal{C}_{12,10}$	FIWWPGGAPWFWWWFWWWWAGPPWGWLFF
$\mathcal{C}_{12,11}$	QQWWWWWQWQWWWWWMIIWWQ	$\mathcal{C}_{12,12}$	WYWYYIWWYYYIYRYYYWKYY
$\mathcal{C}_{12,13}$	WPFWWFFWPPFHFFFWWFPWF	$\mathcal{C}_{12,14}$	FWPGWRPYWWWWWPWWPPFWP
$\mathcal{C}_{12,15}$	WFFFRYRWYWFWWWWWDWWWW	$\mathcal{C}_{12,16}$	FFYFFMYFWFYFYFFFYWFFF
$\mathcal{C}_{12,17}$	WWWWWRQWNQWWMWWWRQWWW	$\mathcal{C}_{12,18}$	GHWWPPHWPIWPPLPWPIWIPSFWWLPFFP

Table S6 continued:

$\mathcal{C}_{12,19}$	FWWFWWWFFFFKFFYFWWWWF	$\mathcal{C}_{12,20}$	PPWPPWWWWPWPWWMVWWIPW
$\mathcal{C}_{12,21}$	PWLYWWWPFPWPPIFPWPPPP	$\mathcal{C}_{12,22}$	RIFYFWWPWWHWWPWPFFRYPPFPYWAIW
$\mathcal{C}_{12,23}$	DFHWWWWYWYWWWWFFFWWDWSDI	$\mathcal{C}_{12,24}$	FWTWWPIIFFTWLWLTLPCWFWWP
$\mathcal{C}_{12,25}$	MQTFCWWFQWWWMPWWWPWFW	$\mathcal{C}_{12,26}$	LLPWFTPPPWWWLWWPPWGPP
$\mathcal{C}_{12,27}$	EWWYMWMWQWQWWWWWFWKKK	$\mathcal{C}_{12,28}$	FFFFPHPWWFFFFPFFFWFWW
$\mathcal{C}_{12,29}$	LWPFWWPWPYWPWWPWFPYFP	$\mathcal{C}_{12,30}$	LWDDHWWLLIYLLLFLLRRKW
$\mathcal{C}_{12,31}$	PPIPPPPWWDWPWPWPWKPPR	$\mathcal{C}_{12,32}$	TWDDPWPYTWPYYWWPWPKKK
$\mathcal{C}_{12,33}$	WWQQQQQKQQWQQFYYQWQWW		
$\mathcal{C}_{13,1}$	DDWFDEVTEWPWDWRWEEPYQTRWVNKDWTYHKHRKKRWHDRKYRRRKFQ	$\mathcal{C}_{13,2}$	EFMENQYDWTWEYEHMYWEDWWRPKRFCYYREPCMRHRYWRWRRKKRRYN
$\mathcal{C}_{14,1}$	RWWWVIWHRYNWWRLDWYWSWPYHWYSWYHYWYDVWMPWIYYWDHFWCIS	$\mathcal{C}_{14,2}$	WYLLMNFVDWYYYWYYWMYIYHEYWYWPYWYWFPEFWCWRYYRWRWN
$\mathcal{C}_{14,3}$	HNFGWGWPYPMPHAWFVWIWWFGPMFGWWPASWGWHIFFPGPYSWHFSMW	$\mathcal{C}_{14,4}$	PTWACWPYPWWWFGLWAHVCHPYYWYIWFYLGGPYSPV
$\mathcal{C}_{14,5}$	WFWFWPFPWPYPYWPWPWYPP	$\mathcal{C}_{14,6}$	PPPWYPWWFMWWYFPPPPPPW
$\mathcal{C}_{14,7}$	WGWPIPWWWLWWWWWWAWWAW	$\mathcal{C}_{14,8}$	IWFDWWIMLFFILIDDWWLPWPWPHMMINGWWVWWWPHPFWMGWLYCPGC
$\mathcal{C}_{14,9}$	WWWIWWFHWHWWWFDFRWWWW	$\mathcal{C}_{14,10}$	YYYYWYYYMYLWYYYWSWYYY
$\mathcal{C}_{14,11}$	FIWWPGGAPWFWWWFWWWWAGPPWGWLFF	$\mathcal{C}_{14,12}$	QQWWWWWQWQWWWWWMIIWWQ
$\mathcal{C}_{14,13}$	WPFWWFFWPPFHFFFWWFPWF	$\mathcal{C}_{14,14}$	YAYVPPIASFNWGGGPFWWWPGWYSMVPYYHWMWCWHAWFIPYWMP
$\mathcal{C}_{14,15}$	FWPGWRPYWWWWWPWWPPFWP	$\mathcal{C}_{14,16}$	LWHFGTFGCPPLWVSPPRWGWPLFLIPWSGWHVLFGWPWPWYVWPRMY
$\mathcal{C}_{14,17}$	FFYFFMYFWFYFYFFFYWFFF	$\mathcal{C}_{14,18}$	PFWPCPIFVPPLFFPFLWPLPPLYTASFHWVPFFWFMFILHTLSYLTW
$\mathcal{C}_{14,19}$	GHWWPPHWPIWPPLPWPIWIPSFWWLPFFP	$\mathcal{C}_{14,20}$	FWWFWWWFFFFKFFYFWWWWF
$\mathcal{C}_{14,21}$	PPWPPWWWWPWPWWMVWWIPW	$\mathcal{C}_{14,22}$	PWLYWWWPFPWPPIFPWPPPP
$\mathcal{C}_{14,23}$	RIFYFWWPWWHWWPWPFFRYPPFPYWAIW	$\mathcal{C}_{14,24}$	FFCWPWPLPLPPPLPPFFVWP
$\mathcal{C}_{14,25}$	FWTWWPIIFFTWLWLTLPCWFWWP	$\mathcal{C}_{14,26}$	MQTFCWWFQWWWMPWWWPWFW
$\mathcal{C}_{14,27}$	LLPWFTPPPWWWLWWPPWGPP	$\mathcal{C}_{14,28}$	FFFFPHPWWFFFFPFFFWFWW
$\mathcal{C}_{14,29}$	LWPFWWPWPYWPWWPWFPYFP	$\mathcal{C}_{14,30}$	PPIPPPPWWDWPWPWPWKPPR
$\mathcal{C}_{14,31}$	KWVWNWHRYFHFHVYWLFYWYWRHWYWPWHFFDIWFWLYLLPFYWNCYW	$\mathcal{C}_{14,32}$	DFFWINYGMWWWWVYSLVWWLWLPWVWWFYLYGYDFFWFFYFYWFHYW
$\mathcal{C}_{15,1}$	RWWWVIWHRYNWWRLDWYWSWPYHWYSWYHYWYDVWMPWIYYWDHFWCIS	$\mathcal{C}_{15,2}$	WYLLMNFVDWYYYWYYWMYIYHEYWYWPYWYWFPEFWCWRYYRWRWN
$\mathcal{C}_{15,3}$	WHWYYVMDIHWDFCHWWWYFYEHHILPHKRWWHFWFMRMCLWHQFFWFHR	$\mathcal{C}_{15,4}$	TPSYCWVSSWWMFCQNYLHYDHWDHWYWFYRWWYQWWHRKKYFWWWWPWY
$\mathcal{C}_{15,5}$	WPWWFHWWRRHWWYWYWWFFT	$\mathcal{C}_{15,6}$	DWWDYPMTWDWTMWFYSWWCVWFEYWEWWNWYCFWSLWLFFSIFWVHWW
$\mathcal{C}_{15,7}$	WHHWYTWDWLWMYWWWWWFWD	$\mathcal{C}_{15,8}$	WFWFWPFPWPYPYWPWPWYPP
$\mathcal{C}_{15,9}$	WGWPIPWWWLWWWWWWAWWAW	$\mathcal{C}_{15,10}$	IWFDWWIMLFFILIDDWWLPWPWPHMMINGWWVWWWPHPFWMGWLYCPGC
$\mathcal{C}_{15,11}$	WWWIWWFHWHWWWFDFRWWWW	$\mathcal{C}_{15,12}$	YYYYWYYYMYLWYYYWSWYYY
$\mathcal{C}_{15,13}$	FIWWPGGAPWFWWWFWWWWAGPPWGWLFF	$\mathcal{C}_{15,14}$	QQWWWWWQWQWWWWWMIIWWQ
$\mathcal{C}_{15,15}$	WPFWWFFWPPFHFFFWWFPWF	$\mathcal{C}_{15,16}$	FWPGWRPYWWWWWPWWPPFWP
$\mathcal{C}_{15,17}$	FFYFFMYFWFYFYFFFYWFFF	$\mathcal{C}_{15,18}$	GHWWPPHWPIWPPLPWPIWIPSFWWLPFFP
$\mathcal{C}_{15,19}$	FWWFWWWFFFFKFFYFWWWWF	$\mathcal{C}_{15,20}$	PPWPPWWWWPWPWWMVWWIPW
$\mathcal{C}_{15,21}$	RIFYFWWPWWHWWPWPFFRYPPFPYWAIW	$\mathcal{C}_{15,22}$	FWTWWPIIFFTWLWLTLPCWFWWP
$\mathcal{C}_{15,23}$	MQTFCWWFQWWWMPWWWPWFW	$\mathcal{C}_{15,24}$	FFFFPHPWWFFFFPFFFWFWW
$\mathcal{C}_{15,25}$	LWPFWWPWPYWPWWPWFPYFP	$\mathcal{C}_{15,26}$	LWWNWYWWYFHYFWWNWHMNPHWFYWHWVHYYKYHWYFYRLKWWHWHHWR
$\mathcal{C}_{15,27}$	KWVWNWHRYFHFHVYWLFYWYWRHWYWPWHFFDIWFWLYLLPFYWNCYW	$\mathcal{C}_{15,28}$	DQWLFVDRWVWWRLWKWWKSKWLWWWWWSWPWWWHWYCWYWPVWIYWWY
$\mathcal{C}_{15,29}$	DFFWINYGMWWWWVYSLVWWLWLPWVWWFYLYGYDFFWFFYFYWFHYW		
$\mathcal{C}_{16,1}$	RWWWVIWHRYNWWRLDWYWSWPYHWYSWYHYWYDVWMPWIYYWDHFWCIS	$\mathcal{C}_{16,2}$	WYLLMNFVDWYYYWYYWMYIYHEYWYWPYWYWFPEFWCWRYYRWRWN
$\mathcal{C}_{16,3}$	WPWWFHWWRRHWWYWYWWFFT	$\mathcal{C}_{16,4}$	WHHWYTWDWLWMYWWWWWFWD
$\mathcal{C}_{16,5}$	PTWACWPYPWWWFGLWAHVCHPYYWYIWFYLGGPYSPV	$\mathcal{C}_{16,6}$	WFWFWPFPWPYPYWPWPWYPP
$\mathcal{C}_{16,7}$	PPPWYPWWFMWWYFPPPPPPW	$\mathcal{C}_{16,8}$	WGWPIPWWWLWWWWWWAWWAW
$\mathcal{C}_{16,9}$	WWWIWWFHWHWWWFDFRWWWW	$\mathcal{C}_{16,10}$	YYYYWYYYMYLWYYYWSWYYY
$\mathcal{C}_{16,11}$	WWMYRYWWWWRYWHWFWPYFWWWRW	$\mathcal{C}_{16,12}$	FIWWPGGAPWFWWWFWWWWAGPPWGWLFF
$\mathcal{C}_{16,13}$	QQWWWWWQWQWWWWWMIIWWQ	$\mathcal{C}_{16,14}$	WYWYYIWWYYYIYRYYYWKYY
$\mathcal{C}_{16,15}$	WPFWWFFWPPFHFFFWWFPWF	$\mathcal{C}_{16,16}$	FWPGWRPYWWWWWPWWPPFWP
$\mathcal{C}_{16,17}$	WFFFRYRWYWFWWWWWDWWWW	$\mathcal{C}_{16,18}$	FFYFFMYFWFYFYFFFYWFFF
$\mathcal{C}_{16,19}$	WWWWWRQWNQWWMWWWRQWWW	$\mathcal{C}_{16,20}$	GHWWPPHWPIWPPLPWPIWIPSFWWLPFFP
$\mathcal{C}_{16,21}$	FWWFWWWFFFFKFFYFWWWWF	$\mathcal{C}_{16,22}$	PPWPPWWWWPWPWWMVWWIPW
$\mathcal{C}_{16,23}$	PWLYWWWPFPWPPIFPWPPPP	$\mathcal{C}_{16,24}$	RIFYFWWPWWHWWPWPFFRYPPFPYWAIW

Table S6 continued:

$\mathcal{C}_{16,25}$	DFHWWWWYWYWWWWFFFWWDWSDI	$\mathcal{C}_{16,26}$	FWTWWPIIFFTWLWLTLPCWFWWP
$\mathcal{C}_{16,27}$	MQTFCWWFQWWWMPWWWPWFW	$\mathcal{C}_{16,28}$	LLPWFTPPPWWWLWWPPWGPP
$\mathcal{C}_{16,29}$	FFFFPHPWWFFFFPFFFWFWW	$\mathcal{C}_{16,30}$	LWPFWWPWPYWPWWPWFPYFP
$\mathcal{C}_{16,31}$	KWVWNWHRYFHFHVYWLFYWYWRHWYWPWHFFDIWFWLYLLPFYWNCYW	$\mathcal{C}_{16,32}$	DFFWINYGMWWWWVYSLVWWLWLPWVWWFYLYGYDFFWFFYFYWFHYW
$\mathcal{C}_{17,1}$	WVWIPHQRYDDHHHMNFAVEHHHEFHYHHQHHHHRFLHMHKDRRRH	$\mathcal{C}_{17,2}$	EWDEWQHHDEQLHHHHERWEYQIEFYRTRKWQRCNHHHKHRRHWKNHCRS
$\mathcal{C}_{17,3}$	HHWKDHEHFHHTDHYCWHYEHHPHYLHHYHYGARCHWTDSVKHWYKWKYY	$\mathcal{C}_{17,4}$	HFHGTHHVYWVHFHAEEWPHEHKWCHWNHWQAKMWKHNHKHHWHHHWW
$\mathcal{C}_{17,5}$	AWHWHWHRSFRFEHMYHDYMHYHETDISLWHHVYDFKEHHHRNHHWKKRD	$\mathcal{C}_{17,6}$	FYLHHIDHDVHETTHWRGHHHWHKHCMHHLHHRDSKHKHHMKYFNWHHHR
$\mathcal{C}_{17,7}$	HWHFLQHRHHIYQWWAHIHHHHALRHWHDEHEHKYYGTHKHHVFHHHHFV	$\mathcal{C}_{17,8}$	DHYQVHHHKQYHRHHHTYHVHLHFWWHDYPRWHCHHWHTMYWEHRIHTVH
$\mathcal{C}_{17,9}$	HWEHHSHQDRHHHHHCYRMDHHHLHQWHAIHYDCHHWHHHYKAPKIHWMK	$\mathcal{C}_{17,10}$	DQHYHYDYSWHWNMYEHYHEHHHIQTHLWSHKHHEKHRHRFVWWRRRYRS
$\mathcal{C}_{17,11}$	KHLHVQHPKHHHHRHHYHRYIHLHIHFHHMVHDELHNHITCEHHNHHRVH		
$\mathcal{C}_{18,1}$	RWWWVIWHRYNWWRLDWYWSWPYHWYSWYHYWYDVWMPWIYYWDHFWCIS	$\mathcal{C}_{18,2}$	WYLLMNFVDWYYYWYYWMYIYHEYWYWPYWYWFPEFWCWRYYRWRWN
$\mathcal{C}_{18,3}$	WPWWFHWWRRHWWYWYWWFFT	$\mathcal{C}_{18,4}$	WHHWYTWDWLWMYWWWWWFWD
$\mathcal{C}_{18,5}$	PTWACWPYPWWWFGLWAHVCHPYYWYIWFYLGGPYSPV	$\mathcal{C}_{18,6}$	WFWFWPFPWPYPYWPWPWYPP
$\mathcal{C}_{18,7}$	PPPWYPWWFMWWYFPPPPPPW	$\mathcal{C}_{18,8}$	WGWPIPWWWLWWWWWWAWWAW
$\mathcal{C}_{18,9}$	WWWIWWFHWHWWWFDFRWWWW	$\mathcal{C}_{18,10}$	YYYYWYYYMYLWYYYWSWYYY
$\mathcal{C}_{18,11}$	WWMYRYWWWWRYWHWFWPYFWWWRW	$\mathcal{C}_{18,12}$	FIWWPGGAPWFWWWFWWWWAGPPWGWLFF
$\mathcal{C}_{18,13}$	QQWWWWWQWQWWWWWMIIWWQ	$\mathcal{C}_{18,14}$	WYWYYIWWYYYIYRYYYWKYY
$\mathcal{C}_{18,15}$	WPFWWFFWPPFHFFFWWFPWF	$\mathcal{C}_{18,16}$	FWPGWRPYWWWWWPWWPPFWP
$\mathcal{C}_{18,17}$	WFFFRYRWYWFWWWWWDWWWW	$\mathcal{C}_{18,18}$	FFYFFMYFWFYFYFFFYWFFF
$\mathcal{C}_{18,19}$	WWWWWRQWNQWWMWWWRQWWW	$\mathcal{C}_{18,20}$	GHWWPPHWPIWPPLPWPIWIPSFWWLPFFP
$\mathcal{C}_{18,21}$	FWWFWWWFFFFKFFYFWWWWF	$\mathcal{C}_{18,22}$	PPWPPWWWWPWPWWMVWWIPW
$\mathcal{C}_{18,23}$	PWLYWWWPFPWPPIFPWPPPP	$\mathcal{C}_{18,24}$	RIFYFWWPWWHWWPWPFFRYPPFPYWAIW
$\mathcal{C}_{18,25}$	DFHWWWWYWYWWWWFFFWWDWSDI	$\mathcal{C}_{18,26}$	FWTWWPIIFFTWLWLTLPCWFWWP
$\mathcal{C}_{18,27}$	MQTFCWWFQWWWMPWWWPWFW	$\mathcal{C}_{18,28}$	LLPWFTPPPWWWLWWPPWGPP
$\mathcal{C}_{18,29}$	FFFFPHPWWFFFFPFFFWFWW	$\mathcal{C}_{18,30}$	LWPFWWPWPYWPWWPWFPYFP
$\mathcal{C}_{18,31}$	PPIPPPPWWDWPWPWPWKPPR	$\mathcal{C}_{18,32}$	TWDDPWPYTWPYYWWPWPKKK
$\mathcal{C}_{18,33}$	KWVWNWHRYFHFHVYWLFYWYWRHWYWPWHFFDIWFWLYLLPFYWNCYW	$\mathcal{C}_{18,34}$	DQWLFVDRWVWWRLWKWWKSKWLWWWWWSWPWWWHWYCWYWPVWIYWWY
$\mathcal{C}_{18,35}$	DFFWINYGMWWWWVYSLVWWLWLPWVWWFYLYGYDFFWFFYFYWFHYW		
$\mathcal{C}_{19,1}$	RWWWVIWHRYNWWRLDWYWSWPYHWYSWYHYWYDVWMPWIYYWDHFWCIS	$\mathcal{C}_{19,2}$	WYLLMNFVDWYYYWYYWMYIYHEYWYWPYWYWFPEFWCWRYYRWRWN
$\mathcal{C}_{19,3}$	WHWYYVMDIHWDFCHWWWYFYEHHILPHKRWWHFWFMRMCLWHQFFWFHR	$\mathcal{C}_{19,4}$	TPSYCWVSSWWMFCQNYLHYDHWDHWYWFYRWWYQWWHRKKYFWWWWPWY
$\mathcal{C}_{19,5}$	HNFGWGWPYPMPHAWFVWIWWFGPMFGWWPASWGWHIFFPGPYSWHFSMW	$\mathcal{C}_{19,6}$	IVPPWFVHYVAIIIWMWRWPWLRIAWYFWCVAWIWHHYWKAWCKWMLWNP
$\mathcal{C}_{19,7}$	FWMIWWTTVHHHVTVVWHHPTHWLHGSWPQHTCWHGCHWWYYHTVMHPFH	$\mathcal{C}_{19,8}$	AWILIYIIYFCHLVHPWLSGHWWWWHHWAFCIHPCPPVFLLFFPHSTPHV
$\mathcal{C}_{19,9}$	DWWDYPMTWDWTMWFYSWWCVWFEYWEWWNWYCFWSLWLFFSIFWVHWW	$\mathcal{C}_{19,10}$	PTWACWPYPWWWFGLWAHVCHPYYWYIWFYLGGPYSPV
$\mathcal{C}_{19,11}$	IWFDWWIMLFFILIDDWWLPWPWPHMMINGWWVWWWPHPFWMGWLYCPGC	$\mathcal{C}_{19,12}$	PNWFPPNLLPTSPLWPLCVPLLFFAWVPNFIWYMTVHWLYPPVSWLPHVC
$\mathcal{C}_{19,13}$	LIWWIHHIWFTMTPHFHYHPGWWMVMIPHWCMWIICIAWAMYWWAIFHVL	$\mathcal{C}_{19,14}$	YAYVPPIASFNWGGGPFWWWPGWYSMVPYYHWMWCWHAWFIPYWMP
$\mathcal{C}_{19,15}$	GWMYPPIYPIWWITHYCPPGFYWTVLIWHLIMVIPIIGVCYYPGFIFVYA	$\mathcal{C}_{19,16}$	LWHFGTFGCPPLWVSPPRWGWPLFLIPWSGWHVLFGWPWPWYVWPRMY
$\mathcal{C}_{19,17}$	PFWPCPIFVPPLFFPFLWPLPPLYTASFHWVPFFWFMFILHTLSYLTW	$\mathcal{C}_{19,18}$	LWWNWYWWYFHYFWWNWHMNPHWFYWHWVHYYKYHWYFYRLKWWHWHHWR
$\mathcal{C}_{19,19}$	KWVWNWHRYFHFHVYWLFYWYWRHWYWPWHFFDIWFWLYLLPFYWNCYW	$\mathcal{C}_{19,20}$	LFSWFFWRHWRHWYMHKWHRVWVHHYDFFWFFWWHWWHWYHHWWHFWH
$\mathcal{C}_{19,21}$	DFFWINYGMWWWWVYSLVWWLWLPWVWWFYLYGYDFFWFFYFYWFHYW		
$\mathcal{C}_{20,1}$	RWWWVIWHRYNWWRLDWYWSWPYHWYSWYHYWYDVWMPWIYYWDHFWCIS	$\mathcal{C}_{20,2}$	WYLLMNFVDWYYYWYYWMYIYHEYWYWPYWYWFPEFWCWRYYRWRWN
$\mathcal{C}_{20,3}$	TPSYCWVSSWWMFCQNYLHYDHWDHWYWFYRWWYQWWHRKKYFWWWWPWY	$\mathcal{C}_{20,4}$	HNFGWGWPYPMPHAWFVWIWWFGPMFGWWPASWGWHIFFPGPYSWHFSMW
$\mathcal{C}_{20,5}$	WPWWFHWWRRHWWYWYWWFFT	$\mathcal{C}_{20,6}$	WHHWYTWDWLWMYWWWWWFWD
$\mathcal{C}_{20,7}$	PTWACWPYPWWWFGLWAHVCHPYYWYIWFYLGGPYSPV	$\mathcal{C}_{20,8}$	WFWFWPFPWPYPYWPWPWYPP
$\mathcal{C}_{20,9}$	PPPWYPWWFMWWYFPPPPPPW	$\mathcal{C}_{20,10}$	WGWPIPWWWLWWWWWWAWWAW
$\mathcal{C}_{20,11}$	IWFDWWIMLFFILIDDWWLPWPWPHMMINGWWVWWWPHPFWMGWLYCPGC	$\mathcal{C}_{20,12}$	WWWIWWFHWHWWWFDFRWWWW
$\mathcal{C}_{20,13}$	YYYYWYYYMYLWYYYWSWYYY	$\mathcal{C}_{20,14}$	FIWWPGGAPWFWWWFWWWWAGPPWGWLFF
$\mathcal{C}_{20,15}$	QQWWWWWQWQWWWWWMIIWWQ	$\mathcal{C}_{20,16}$	WPFWWFFWPPFHFFFWWFPWF
$\mathcal{C}_{20,17}$	YAYVPPIASFNWGGGPFWWWPGWYSMVPYYHWMWCWHAWFIPYWMP	$\mathcal{C}_{20,18}$	FWPGWRPYWWWWWPWWPPFWP
$\mathcal{C}_{20,19}$	LWHFGTFGCPPLWVSPPRWGWPLFLIPWSGWHVLFGWPWPWYVWPRMY	$\mathcal{C}_{20,20}$	FFYFFMYFWFYFYFFFYWFFF
$\mathcal{C}_{20,21}$	GHWWPPHWPIWPPLPWPIWIPSFWWLPFFP	$\mathcal{C}_{20,22}$	FWWFWWWFFFFKFFYFWWWWF
$\mathcal{C}_{20,23}$	PPWPPWWWWPWPWWMVWWIPW	$\mathcal{C}_{20,24}$	PWLYWWWPFPWPPIFPWPPPP
$\mathcal{C}_{20,25}$	RIFYFWWPWWHWWPWPFFRYPPFPYWAIW	$\mathcal{C}_{20,26}$	FWTWWPIIFFTWLWLTLPCWFWWP

Table S6 continued:

$\mathcal{C}_{20,27}$	MQTFCWWFQWWWMPWWWPWFW	$\mathcal{C}_{20,28}$	LLPWFTPPPWWWLWWPPWGPP
$\mathcal{C}_{20,29}$	FFFFPHPWWFFFFPFFFWFWW	$\mathcal{C}_{20,30}$	LWPFWWPWPYWPWWPWFPYFP
$\mathcal{C}_{20,31}$	KWVWNWHRYFHFHVYWLFYWYWRHWYWPWHFFDIWFWLYLLPFYWNCYW	$\mathcal{C}_{20,32}$	DQWLFVDRWVWWRLWKWWKSKWLWWWWWSWPWWWHWYCWYWPVWIYWWY
$\mathcal{C}_{20,33}$	DFFWINYGMWWWWVYSLVWWLWLPWVWWFYLYGYDFFWFFYFYWFHYW		
$\mathcal{C}_{21,1}$	RWWWVIWHRYNWWRLDWYWSWPYHWYSWYHYWYDVWMPWIYYWDHFWCIS	$\mathcal{C}_{21,2}$	WYLLMNFVDWYYYWYYWMYIYHEYWYWPYWYWFPEFWCWRYYRWRWN
$\mathcal{C}_{21,3}$	TPSYCWVSSWWMFCQNYLHYDHWDHWYWFYRWWYQWWHRKKYFWWWWPWY	$\mathcal{C}_{21,4}$	HNFGWGWPYPMPHAWFVWIWWFGPMFGWWPASWGWHIFFPGPYSWHFSMW
$\mathcal{C}_{21,5}$	IVPPWFVHYVAIIIWMWRWPWLRIAWYFWCVAWIWHHYWKAWCKWMLWNP	$\mathcal{C}_{21,6}$	AWILIYIIYFCHLVHPWLSGHWWWWHHWAFCIHPCPPVFLLFFPHSTPHV
$\mathcal{C}_{21,7}$	DWWDYPMTWDWTMWFYSWWCVWFEYWEWWNWYCFWSLWLFFSIFWVHWW	$\mathcal{C}_{21,8}$	PTWACWPYPWWWFGLWAHVCHPYYWYIWFYLGGPYSPV
$\mathcal{C}_{21,9}$	WFWFWPFPWPYPYWPWPWYPP	$\mathcal{C}_{21,10}$	IWFDWWIMLFFILIDDWWLPWPWPHMMINGWWVWWWPHPFWMGWLYCPGC
$\mathcal{C}_{21,11}$	PNWFPPNLLPTSPLWPLCVPLLFFAWVPNFIWYMTVHWLYPPVSWLPHVC	$\mathcal{C}_{21,12}$	FIWWPGGAPWFWWWFWWWWAGPPWGWLFF
$\mathcal{C}_{21,13}$	LIWWIHHIWFTMTPHFHYHPGWWMVMIPHWCMWIICIAWAMYWWAIFHVL	$\mathcal{C}_{21,14}$	YAYVPPIASFNWGGGPFWWWPGWYSMVPYYHWMWCWHAWFIPYWMP
$\mathcal{C}_{21,15}$	GWMYPPIYPIWWITHYCPPGFYWTVLIWHLIMVIPIIGVCYYPGFIFVYA	$\mathcal{C}_{21,16}$	LWHFGTFGCPPLWVSPPRWGWPLFLIPWSGWHVLFGWPWPWYVWPRMY
$\mathcal{C}_{21,17}$	SCYRPPIFYFPWRRRPLWWVWWYWYTTGYIRWLWYHHAWWALSWPP	$\mathcal{C}_{21,18}$	PFWPCPIFVPPLFFPFLWPLPPLYTASFHWVPFFWFMFILHTLSYLTW
$\mathcal{C}_{21,19}$	GHWWPPHWPIWPPLPWPIWIPSFWWLPFFP	$\mathcal{C}_{21,20}$	PPWPPWWWWPWPWWMVWWIPW
$\mathcal{C}_{21,21}$	RIFYFWWPWWHWWPWPFFRYPPFPYWAIW	$\mathcal{C}_{21,22}$	FWTWWPIIFFTWLWLTLPCWFWWP
$\mathcal{C}_{21,23}$	LWPFWWPWPYWPWWPWFPYFP	$\mathcal{C}_{21,24}$	LWWNWYWWYFHYFWWNWHMNPHWFYWHWVHYYKYHWYFYRLKWWHWHHWR
$\mathcal{C}_{21,25}$	KWVWNWHRYFHFHVYWLFYWYWRHWYWPWHFFDIWFWLYLLPFYWNCYW	$\mathcal{C}_{21,26}$	DQWLFVDRWVWWRLWKWWKSKWLWWWWWSWPWWWHWYCWYWPVWIYWWY
$\mathcal{C}_{21,27}$	DFFWINYGMWWWWVYSLVWWLWLPWVWWFYLYGYDFFWFFYFYWFHYW		
$\mathcal{C}_{22,1}$	RWWWVIWHRYNWWRLDWYWSWPYHWYSWYHYWYDVWMPWIYYWDHFWCIS	$\mathcal{C}_{22,2}$	WYLLMNFVDWYYYWYYWMYIYHEYWYWPYWYWFPEFWCWRYYRWRWN
$\mathcal{C}_{22,3}$	TPSYCWVSSWWMFCQNYLHYDHWDHWYWFYRWWYQWWHRKKYFWWWWPWY	$\mathcal{C}_{22,4}$	WPWWFHWWRRHWWYWYWWFFT
$\mathcal{C}_{22,5}$	WHHWYTWDWLWMYWWWWWFWD	$\mathcal{C}_{22,6}$	PTWACWPYPWWWFGLWAHVCHPYYWYIWFYLGGPYSPV
$\mathcal{C}_{22,7}$	WFWFWPFPWPYPYWPWPWYPP	$\mathcal{C}_{22,8}$	PPPWYPWWFMWWYFPPPPPPW
$\mathcal{C}_{22,9}$	WGWPIPWWWLWWWWWWAWWAW	$\mathcal{C}_{22,10}$	WWWIWWFHWHWWWFDFRWWWW
$\mathcal{C}_{22,11}$	YYYYWYYYMYLWYYYWSWYYY	$\mathcal{C}_{22,12}$	WWMYRYWWWWRYWHWFWPYFWWWRW
$\mathcal{C}_{22,13}$	FIWWPGGAPWFWWWFWWWWAGPPWGWLFF	$\mathcal{C}_{22,14}$	QQWWWWWQWQWWWWWMIIWWQ
$\mathcal{C}_{22,15}$	WYWYYIWWYYYIYRYYYWKYY	$\mathcal{C}_{22,16}$	WPFWWFFWPPFHFFFWWFPWF
$\mathcal{C}_{22,17}$	FWPGWRPYWWWWWPWWPPFWP	$\mathcal{C}_{22,18}$	WFFFRYRWYWFWWWWWDWWWW
$\mathcal{C}_{22,19}$	FFYFFMYFWFYFYFFFYWFFF	$\mathcal{C}_{22,20}$	WWWWWRQWNQWWMWWWRQWWW
$\mathcal{C}_{22,21}$	GHWWPPHWPIWPPLPWPIWIPSFWWLPFFP	$\mathcal{C}_{22,22}$	FWWFWWWFFFFKFFYFWWWWF
$\mathcal{C}_{22,23}$	PPWPPWWWWPWPWWMVWWIPW	$\mathcal{C}_{22,24}$	PWLYWWWPFPWPPIFPWPPPP
$\mathcal{C}_{22,25}$	RIFYFWWPWWHWWPWPFFRYPPFPYWAIW	$\mathcal{C}_{22,26}$	DFHWWWWYWYWWWWFFFWWDWSDI
$\mathcal{C}_{22,27}$	FWTWWPIIFFTWLWLTLPCWFWWP	$\mathcal{C}_{22,28}$	MQTFCWWFQWWWMPWWWPWFW
$\mathcal{C}_{22,29}$	FFFFPHPWWFFFFPFFFWFWW	$\mathcal{C}_{22,30}$	LWPFWWPWPYWPWWPWFPYFP
$\mathcal{C}_{22,31}$	KWVWNWHRYFHFHVYWLFYWYWRHWYWPWHFFDIWFWLYLLPFYWNCYW	$\mathcal{C}_{22,32}$	DQWLFVDRWVWWRLWKWWKSKWLWWWWWSWPWWWHWYCWYWPVWIYWWY
$\mathcal{C}_{22,33}$	DFFWINYGMWWWWVYSLVWWLWLPWVWWFYLYGYDFFWFFYFYWFHYW		
$\mathcal{C}_{23,1}$	RWWWVIWHRYNWWRLDWYWSWPYHWYSWYHYWYDVWMPWIYYWDHFWCIS	$\mathcal{C}_{23,2}$	EWMWWKVWYYTDWWRWWRDFRCWWLRWWFRRHPYYLWWWYWYIWHWWHDR
$\mathcal{C}_{23,3}$	WYLLMNFVDWYYYWYYWMYIYHEYWYWPYWYWFPEFWCWRYYRWRWN	$\mathcal{C}_{23,4}$	TPSYCWVSSWWMFCQNYLHYDHWDHWYWFYRWWYQWWHRKKYFWWWWPWY
$\mathcal{C}_{23,5}$	DWWDYPMTWDWTMWFYSWWCVWFEYWEWWNWYCFWSLWLFFSIFWVHWW	$\mathcal{C}_{23,6}$	WHHWYTWDWLWMYWWWWWFWD
$\mathcal{C}_{23,7}$	IWFDWWIMLFFILIDDWWLPWPWPHMMINGWWVWWWPHPFWMGWLYCPGC	$\mathcal{C}_{23,8}$	WWWIWWFHWHWWWFDFRWWWW
$\mathcal{C}_{23,9}$	WWMYRYWWWWRYWHWFWPYFWWWRW	$\mathcal{C}_{23,10}$	FWWFWWWFFFFKFFYFWWWWF
$\mathcal{C}_{23,11}$	RIFYFWWPWWHWWPWPFFRYPPFPYWAIW	$\mathcal{C}_{23,12}$	DFHWWWWYWYWWWWFFFWWDWSDI
$\mathcal{C}_{23,13}$	LWWNWYWWYFHYFWWNWHMNPHWFYWHWVHYYKYHWYFYRLKWWHWHHWR	$\mathcal{C}_{23,14}$	KWVWNWHRYFHFHVYWLFYWYWRHWYWPWHFFDIWFWLYLLPFYWNCYW
$\mathcal{C}_{23,15}$	DQWLFVDRWVWWRLWKWWKSKWLWWWWWSWPWWWHWYCWYWPVWIYWWY	$\mathcal{C}_{23,16}$	DFFWINYGMWWWWVYSLVWWLWLPWVWWFYLYGYDFFWFFYFYWFHYW
$\mathcal{C}_{24,1}$	RWWWVIWHRYNWWRLDWYWSWPYHWYSWYHYWYDVWMPWIYYWDHFWCIS	$\mathcal{C}_{24,2}$	WYLLMNFVDWYYYWYYWMYIYHEYWYWPYWYWFPEFWCWRYYRWRWN
$\mathcal{C}_{24,3}$	TPSYCWVSSWWMFCQNYLHYDHWDHWYWFYRWWYQWWHRKKYFWWWWPWY	$\mathcal{C}_{24,4}$	WPWWFHWWRRHWWYWYWWFFT
$\mathcal{C}_{24,5}$	DWWDYPMTWDWTMWFYSWWCVWFEYWEWWNWYCFWSLWLFFSIFWVHWW	$\mathcal{C}_{24,6}$	WHHWYTWDWLWMYWWWWWFWD
$\mathcal{C}_{24,7}$	WFWFWPFPWPYPYWPWPWYPP	$\mathcal{C}_{24,8}$	WGWPIPWWWLWWWWWWAWWAW
$\mathcal{C}_{24,9}$	IWFDWWIMLFFILIDDWWLPWPWPHMMINGWWVWWWPHPFWMGWLYCPGC	$\mathcal{C}_{24,10}$	WWWIWWFHWHWWWFDFRWWWW
$\mathcal{C}_{24,11}$	YYYYWYYYMYLWYYYWSWYYY	$\mathcal{C}_{24,12}$	WWMYRYWWWWRYWHWFWPYFWWWRW
$\mathcal{C}_{24,13}$	FIWWPGGAPWFWWWFWWWWAGPPWGWLFF	$\mathcal{C}_{24,14}$	QQWWWWWQWQWWWWWMIIWWQ
$\mathcal{C}_{24,15}$	WYWYYIWWYYYIYRYYYWKYY	$\mathcal{C}_{24,16}$	WPFWWFFWPPFHFFFWWFPWF
$\mathcal{C}_{24,17}$	FWPGWRPYWWWWWPWWPPFWP	$\mathcal{C}_{24,18}$	WFFFRYRWYWFWWWWWDWWWW

Table S6 continued:

$\mathcal{C}_{24,19}$	FFYFFMYFWFYFYFFFYWFFF	$\mathcal{C}_{24,20}$	WWWWWRQWNQWWMWWWRQWWW
$\mathcal{C}_{24,21}$	GHWWPPHWPIWPPLPWPIWIPSFWWLPFFP	$\mathcal{C}_{24,22}$	FWWFWWWFFFFKFFYFWWWWF
$\mathcal{C}_{24,23}$	PPWPPWWWWPWPWWMVWWIPW	$\mathcal{C}_{24,24}$	RIFYFWWPWWHWWPWPFFRYPPFPYWAIW
$\mathcal{C}_{24,25}$	DFHWWWWYWYWWWWFFFWWDWSDI	$\mathcal{C}_{24,26}$	FWTWWPIIFFTWLWLTLPCWFWWP
$\mathcal{C}_{24,27}$	MQTFCWWFQWWWMPWWWPWFW	$\mathcal{C}_{24,28}$	FFFFPHPWWFFFFPFFFWFWW
$\mathcal{C}_{24,29}$	LWPFWWPWPYWPWWPWFPYFP	$\mathcal{C}_{24,30}$	LWWNWYWWYFHYFWWNWHMNPHWFYWHWVHYYKYHWYFYRLKWWHWHHWR
$\mathcal{C}_{24,31}$	KWVWNWHRYFHFHVYWLFYWYWRHWYWPWHFFDIWFWLYLLPFYWNCYW	$\mathcal{C}_{24,32}$	DQWLFVDRWVWWRLWKWWKSKWLWWWWWSWPWWWHWYCWYWPVWIYWWY
$\mathcal{C}_{24,33}$	DFFWINYGMWWWWVYSLVWWLWLPWVWWFYLYGYDFFWFFYFYWFHYW		
$\mathcal{C}_{25,1}$	RWWWVIWHRYNWWRLDWYWSWPYHWYSWYHYWYDVWMPWIYYWDHFWCIS	$\mathcal{C}_{25,2}$	EWMWWKVWYYTDWWRWWRDFRCWWLRWWFRRHPYYLWWWYWYIWHWWHDR
$\mathcal{C}_{25,3}$	WYLLMNFVDWYYYWYYWMYIYHEYWYWPYWYWFPEFWCWRYYRWRWN	$\mathcal{C}_{25,4}$	WHWYYVMDIHWDFCHWWWYFYEHHILPHKRWWHFWFMRMCLWHQFFWFHR
$\mathcal{C}_{25,5}$	TPSYCWVSSWWMFCQNYLHYDHWDHWYWFYRWWYQWWHRKKYFWWWWPWY	$\mathcal{C}_{25,6}$	IVPPWFVHYVAIIIWMWRWPWLRIAWYFWCVAWIWHHYWKAWCKWMLWNP
$\mathcal{C}_{25,7}$	DWWDYPMTWDWTMWFYSWWCVWFEYWEWWNWYCFWSLWLFFSIFWVHWW	$\mathcal{C}_{25,8}$	WHHWYTWDWLWMYWWWWWFWD
$\mathcal{C}_{25,9}$	WGWPIPWWWLWWWWWWAWWAW	$\mathcal{C}_{25,10}$	IWFDWWIMLFFILIDDWWLPWPWPHMMINGWWVWWWPHPFWMGWLYCPGC
$\mathcal{C}_{25,11}$	FIWWPGGAPWFWWWFWWWWAGPPWGWLFF	$\mathcal{C}_{25,12}$	FWPGWRPYWWWWWPWWPPFWP
$\mathcal{C}_{25,13}$	SCYRPPIFYFPWRRRPLWWVWWYWYTTGYIRWLWYHHAWWALSWPP	$\mathcal{C}_{25,14}$	FWWFWWWFFFFKFFYFWWWWF
$\mathcal{C}_{25,15}$	RIFYFWWPWWHWWPWPFFRYPPFPYWAIW	$\mathcal{C}_{25,16}$	DFHWWWWYWYWWWWFFFWWDWSDI
$\mathcal{C}_{25,17}$	LWWNWYWWYFHYFWWNWHMNPHWFYWHWVHYYKYHWYFYRLKWWHWHHWR	$\mathcal{C}_{25,18}$	KWVWNWHRYFHFHVYWLFYWYWRHWYWPWHFFDIWFWLYLLPFYWNCYW
$\mathcal{C}_{25,19}$	DQWLFVDRWVWWRLWKWWKSKWLWWWWWSWPWWWHWYCWYWPVWIYWWY	$\mathcal{C}_{25,20}$	DFFWINYGMWWWWVYSLVWWLWLPWVWWFYLYGYDFFWFFYFYWFHYW
$\mathcal{C}_{26,1}$	HWWWNDWRDWWYHDVYFYIYNWKIWYSHYFWYYRWRNRWIKPWDRKYMPE	$\mathcal{C}_{26,2}$	EPFYMFYWDMYYYPDTYFYWHMWIMWCLFDVYYMHQWQITWFWKRKRKYK
$\mathcal{C}_{26,3}$	RWWWVIWHRYNWWRLDWYWSWPYHWYSWYHYWYDVWMPWIYYWDHFWCIS	$\mathcal{C}_{26,4}$	EWMWWKVWYYTDWWRWWRDFRCWWLRWWFRRHPYYLWWWYWYIWHWWHDR
$\mathcal{C}_{26,5}$	WYLLMNFVDWYYYWYYWMYIYHEYWYWPYWYWFPEFWCWRYYRWRWN	$\mathcal{C}_{26,6}$	WHWYYVMDIHWDFCHWWWYFYEHHILPHKRWWHFWFMRMCLWHQFFWFHR
$\mathcal{C}_{26,7}$	TPSYCWVSSWWMFCQNYLHYDHWDHWYWFYRWWYQWWHRKKYFWWWWPWY	$\mathcal{C}_{26,8}$	WPWWFHWWRRHWWYWYWWFFT
$\mathcal{C}_{26,9}$	DWWDYPMTWDWTMWFYSWWCVWFEYWEWWNWYCFWSLWLFFSIFWVHWW	$\mathcal{C}_{26,10}$	WHHWYTWDWLWMYWWWWWFWD
$\mathcal{C}_{26,11}$	WFWFWPFPWPYPYWPWPWYPP	$\mathcal{C}_{26,12}$	WGWPIPWWWLWWWWWWAWWAW
$\mathcal{C}_{26,13}$	IWFDWWIMLFFILIDDWWLPWPWPHMMINGWWVWWWPHPFWMGWLYCPGC	$\mathcal{C}_{26,14}$	WWWIWWFHWHWWWFDFRWWWW
$\mathcal{C}_{26,15}$	YYYYWYYYMYLWYYYWSWYYY	$\mathcal{C}_{26,16}$	WWMYRYWWWWRYWHWFWPYFWWWRW
$\mathcal{C}_{26,17}$	FIWWPGGAPWFWWWFWWWWAGPPWGWLFF	$\mathcal{C}_{26,18}$	QQWWWWWQWQWWWWWMIIWWQ
$\mathcal{C}_{26,19}$	WYWYYIWWYYYIYRYYYWKYY	$\mathcal{C}_{26,20}$	WPFWWFFWPPFHFFFWWFPWF
$\mathcal{C}_{26,21}$	FWPGWRPYWWWWWPWWPPFWP	$\mathcal{C}_{26,22}$	WFFFRYRWYWFWWWWWDWWWW
$\mathcal{C}_{26,23}$	FFYFFMYFWFYFYFFFYWFFF	$\mathcal{C}_{26,24}$	FWWFWWWFFFFKFFYFWWWWF
$\mathcal{C}_{26,25}$	PPWPPWWWWPWPWWMVWWIPW	$\mathcal{C}_{26,26}$	RIFYFWWPWWHWWPWPFFRYPPFPYWAIW
$\mathcal{C}_{26,27}$	DFHWWWWYWYWWWWFFFWWDWSDI	$\mathcal{C}_{26,28}$	MQTFCWWFQWWWMPWWWPWFW
$\mathcal{C}_{26,29}$	FFFFPHPWWFFFFPFFFWFWW	$\mathcal{C}_{26,30}$	LWPFWWPWPYWPWWPWFPYFP
$\mathcal{C}_{26,31}$	LWWNWYWWYFHYFWWNWHMNPHWFYWHWVHYYKYHWYFYRLKWWHWHHWR	$\mathcal{C}_{26,32}$	KWVWNWHRYFHFHVYWLFYWYWRHWYWPWHFFDIWFWLYLLPFYWNCYW
$\mathcal{C}_{26,33}$	DQWLFVDRWVWWRLWKWWKSKWLWWWWWSWPWWWHWYCWYWPVWIYWWY	$\mathcal{C}_{26,34}$	DFFWINYGMWWWWVYSLVWWLWLPWVWWFYLYGYDFFWFFYFYWFHYW
$\mathcal{C}_{27,1}$	RWWWVIWHRYNWWRLDWYWSWPYHWYSWYHYWYDVWMPWIYYWDHFWCIS	$\mathcal{C}_{27,2}$	EWMWWKVWYYTDWWRWWRDFRCWWLRWWFRRHPYYLWWWYWYIWHWWHDR
$\mathcal{C}_{27,3}$	WYLLMNFVDWYYYWYYWMYIYHEYWYWPYWYWFPEFWCWRYYRWRWN	$\mathcal{C}_{27,4}$	TPSYCWVSSWWMFCQNYLHYDHWDHWYWFYRWWYQWWHRKKYFWWWWPWY
$\mathcal{C}_{27,5}$	WPWWFHWWRRHWWYWYWWFFT	$\mathcal{C}_{27,6}$	DWWDYPMTWDWTMWFYSWWCVWFEYWEWWNWYCFWSLWLFFSIFWVHWW
$\mathcal{C}_{27,7}$	WHHWYTWDWLWMYWWWWWFWD	$\mathcal{C}_{27,8}$	WFWFWPFPWPYPYWPWPWYPP
$\mathcal{C}_{27,9}$	WGWPIPWWWLWWWWWWAWWAW	$\mathcal{C}_{27,10}$	WWWIWWFHWHWWWFDFRWWWW
$\mathcal{C}_{27,11}$	YYYYWYYYMYLWYYYWSWYYY	$\mathcal{C}_{27,12}$	WWMYRYWWWWRYWHWFWPYFWWWRW
$\mathcal{C}_{27,13}$	FIWWPGGAPWFWWWFWWWWAGPPWGWLFF	$\mathcal{C}_{27,14}$	QQWWWWWQWQWWWWWMIIWWQ
$\mathcal{C}_{27,15}$	WYWYYIWWYYYIYRYYYWKYY	$\mathcal{C}_{27,16}$	WPFWWFFWPPFHFFFWWFPWF
$\mathcal{C}_{27,17}$	FWPGWRPYWWWWWPWWPPFWP	$\mathcal{C}_{27,18}$	WFFFRYRWYWFWWWWWDWWWW
$\mathcal{C}_{27,19}$	FFYFFMYFWFYFYFFFYWFFF	$\mathcal{C}_{27,20}$	WWWWWRQWNQWWMWWWRQWWW
$\mathcal{C}_{27,21}$	FWWFWWWFFFFKFFYFWWWWF	$\mathcal{C}_{27,22}$	PPWPPWWWWPWPWWMVWWIPW
$\mathcal{C}_{27,23}$	RIFYFWWPWWHWWPWPFFRYPPFPYWAIW	$\mathcal{C}_{27,24}$	DFHWWWWYWYWWWWFFFWWDWSDI
$\mathcal{C}_{27,25}$	MQTFCWWFQWWWMPWWWPWFW	$\mathcal{C}_{27,26}$	FFFFPHPWWFFFFPFFFWFWW
$\mathcal{C}_{27,27}$	LWPFWWPWPYWPWWPWFPYFP	$\mathcal{C}_{27,28}$	LWWNWYWWYFHYFWWNWHMNPHWFYWHWVHYYKYHWYFYRLKWWHWHHWR
$\mathcal{C}_{27,29}$	KWVWNWHRYFHFHVYWLFYWYWRHWYWPWHFFDIWFWLYLLPFYWNCYW	$\mathcal{C}_{27,30}$	DQWLFVDRWVWWRLWKWWKSKWLWWWWWSWPWWWHWYCWYWPVWIYWWY
$\mathcal{C}_{27,31}$	DFFWINYGMWWWWVYSLVWWLWLPWVWWFYLYGYDFFWFFYFYWFHYW		
$\mathcal{C}_{28,1}$	HWWWNDWRDWWYHDVYFYIYNWKIWYSHYFWYYRWRNRWIKPWDRKYMPE	$\mathcal{C}_{28,2}$	EPFYMFYWDMYYYPDTYFYWHMWIMWCLFDVYYMHQWQITWFWKRKRKYK

Table S6 continued:

$\mathcal{C}_{28,3}$	RWWWVIWHRYNWWRLDWYWSWPYHWYSWYHYWYDVWMPWIYYWDHFWCIS	$\mathcal{C}_{28,4}$	EWMWWKVWYYTDWWRWWRDFRCWWLRWWFRRHPYYLWWWYWYIWHWWHDR
$\mathcal{C}_{28,5}$	WYLLMNFVDWYYYWYYWMYIYHEYWYWPYWYWFPEFWCWRYYRWRWN	$\mathcal{C}_{28,6}$	WHWYYYVMDIHWDFCHWWWYFYEHHILPHKRWWHFWFMRMCLWHQFFWFHR
$\mathcal{C}_{28,7}$	TPSYCWVSSWWMFCQNYLHYDHWDHWYWFYRWWYQWWHRKKYFWWWWPWY	$\mathcal{C}_{28,8}$	IVPPWFVHYVAIIIWMWRWPWLRIAWYFWCVAWIWHHYWKAWCKWMLWNP
$\mathcal{C}_{28,9}$	FWMIWWTTVHHHVTVVWHHPTHWLHGSWPQHTCWHGCHWWYYHTVMHPFH	$\mathcal{C}_{28,10}$	AWILIYIIYFCHLVHPWLSGHWWWWHHWAFCIHPCPPVFLLFFPHSTPHV
$\mathcal{C}_{28,11}$	DWWDYPMTWDWTMWFYSWWCVWFEYWEWWNWYCFWSLWLFFSIFWVHWW	$\mathcal{C}_{28,12}$	IWFDWWIMLFFILIDDWWLPWPWPHMMINGWWVWWWPHPFWMGWLYCPGC
$\mathcal{C}_{28,13}$	LIWWIHHIWFTMTPHFHYHPGWWMVMIPHWCMWIICIAWAMYWWAIFHVL	$\mathcal{C}_{28,14}$	SCYRPPIFYFPWRRRPLWWVWWYWYTTGYIRWLWYHHAWWALSWPP
$\mathcal{C}_{28,15}$	LWWNWYWWYFHYFWWNWHMNPHWFYWHWVHYYKYHWYFYRLKWWHWHHWR	$\mathcal{C}_{28,16}$	KWVWNWHRYFHFHVYWLFYWYWRHWYWPWHFFDIWFWLYLLPFYWNCYW
$\mathcal{C}_{28,17}$	LFSWFFWRHWRHWYMHKWHRVWVHHYDFFWFFWWHWWHWYHHWWHFWH	$\mathcal{C}_{28,18}$	DQWLFVDRWVWWRLWKWWKSKWLWWWWWWSWPWWWHWYCWYWPVWIYWWY
$\mathcal{C}_{28,19}$	DFFWINYGMWWWWVYSLVWWLWLPWVWWFYLYGYDFFWFFYFYWFHYW		
$\mathcal{C}_{29,1}$	EPFYMFYWDMYYYPDTYFYWHMWIMWCLFDVYYMHQWQITWFWKRKRKYK	$\mathcal{C}_{29,2}$	RWWWVIWHRYNWWRLDWYWSWPYHWYSWYHYWYDVWMPWIYYWDHFWCIS
$\mathcal{C}_{29,3}$	WYLLMNFVDWYYYWYYWMYIYHEYWYWPYWYWFPEFWCWRYYRWRWN	$\mathcal{C}_{29,4}$	WHWYYYVMDIHWDFCHWWWYFYEHHILPHKRWWHFWFMRMCLWHQFFWFHR
$\mathcal{C}_{29,5}$	TPSYCWVSSWWMFCQNYLHYDHWDHWYWFYRWWYQWWHRKKYFWWWWPWY	$\mathcal{C}_{29,6}$	HNFGWGWPYPMPHAWFVWIWWFGPMFGWWPASWGWHIFFPGPYSWHFSMW
$\mathcal{C}_{29,7}$	IVPPWFVHYVAIIIWMWRWPWLRIAWYFWCVAWIWHHYWKAWCKWMLWNP	$\mathcal{C}_{29,8}$	AWILIYIIYFCHLVHPWLSGHWWWWHHWAFCIHPCPPVFLLFFPHSTPHV
$\mathcal{C}_{29,9}$	DWWDYPMTWDWTMWFYSWWCVWFEYWEWWNWYCFWSLWLFFSIFWVHWW	$\mathcal{C}_{29,10}$	PTWACWPYPWWWFGLWAHVCHPYYWYIWFYLGGPYSPV
$\mathcal{C}_{29,11}$	IWFDWWIMLFFILIDDWWLPWPWPHMMINGWWVWWWPHPFWMGWLYCPGC	$\mathcal{C}_{29,12}$	PNWFPPNLLPTSPLWPLCVPLLFFAWVPNFIWYMTVHWLYPPVSWLPHVC
$\mathcal{C}_{29,13}$	FIWWPGGAPWFWWWFWWWWAGPPWGWLFF	$\mathcal{C}_{29,14}$	LIWWIHHIWFTMTPHFHYHPGWWMVMIPHWCMWIICIAWAMYWWAIFHVL
$\mathcal{C}_{29,15}$	YAYVPPIASFNWGGGPFWWWPGWYSMVPYYHWMWCWHAWFIPYWMP	$\mathcal{C}_{29,16}$	GWMYPPIYPIWWITHYCPPGFYWTVLIWHLIMVIPIIGVCYYPGFIFVYA
$\mathcal{C}_{29,17}$	LWHFGTFGCPPLWVSPPRWGWPLFLIPWSGWHVLFGWPWPWYVWPRMY	$\mathcal{C}_{29,18}$	SCYRPPIFYFPWRRRPLWWVWWYWYTTGYIRWLWYHHAWWALSWPP
$\mathcal{C}_{29,19}$	PFWPCPIFVPPLFFPFLWPLPPLYTASFHWVPFFWFMFILHTLSYLTW	$\mathcal{C}_{29,20}$	GHWWPPHWPIWPPLPWPIWIPSFWWLPFFP
$\mathcal{C}_{29,21}$	RIFYFWWPWWHWWPWPFFRYPPFPYWAIW	$\mathcal{C}_{29,22}$	LWWNWYWWYFHYFWWNWHMNPHWFYWHWVHYYKYHWYFYRLKWWHWHHWR
$\mathcal{C}_{29,23}$	KWVWNWHRYFHFHVYWLFYWYWRHWYWPWHFFDIWFWLYLLPFYWNCYW	$\mathcal{C}_{29,24}$	LFSWFFWRHWRHWYMHKWHRVWVHHYDFFWFFWWHWWHWYHHWWHFWH
$\mathcal{C}_{29,25}$	DQWLFVDRWVWWRLWKWWKSKWLWWWWWWSWPWWWHWYCWYWPVWIYWWY	$\mathcal{C}_{29,26}$	DFFWINYGMWWWWVYSLVWWLWLPWVWWFYLYGYDFFWFFYFYWFHYW
$\mathcal{C}_{30,1}$	HWWWNDWRDWWYHDVYFYIYNWKIWYSHYFWYYRWRNRWIKPWDRKYMPE	$\mathcal{C}_{30,2}$	EPFYMFYWDMYYYPDTYFYWHMWIMWCLFDVYYMHQWQITWFWKRKRKYK
$\mathcal{C}_{30,3}$	RWWWVIWHRYNWWRLDWYWSWPYHWYSWYHYWYDVWMPWIYYWDHFWCIS	$\mathcal{C}_{30,4}$	EWMWWKVWYYTDWWRWWRDFRCWWLRWWFRRHPYYLWWWYWYIWHWWHDR
$\mathcal{C}_{30,5}$	WYLLMNFVDWYYYWYYWMYIYHEYWYWPYWYWFPEFWCWRYYRWRWN	$\mathcal{C}_{30,6}$	WHWYYYVMDIHWDFCHWWWYFYEHHILPHKRWWHFWFMRMCLWHQFFWFHR
$\mathcal{C}_{30,7}$	TPSYCWVSSWWMFCQNYLHYDHWDHWYWFYRWWYQWWHRKKYFWWWWPWY	$\mathcal{C}_{30,8}$	HNFGWGWPYPMPHAWFVWIWWFGPMFGWWPASWGWHIFFPGPYSWHFSMW
$\mathcal{C}_{30,9}$	IVPPWFVHYVAIIIWMWRWPWLRIAWYFWCVAWIWHHYWKAWCKWMLWNP	$\mathcal{C}_{30,10}$	AWILIYIIYFCHLVHPWLSGHWWWWHHWAFCIHPCPPVFLLFFPHSTPHV
$\mathcal{C}_{30,11}$	DWWDYPMTWDWTMWFYSWWCVWFEYWEWWNWYCFWSLWLFFSIFWVHWW	$\mathcal{C}_{30,12}$	PTWACWPYPWWWFGLWAHVCHPYYWYIWFYLGGPYSPV
$\mathcal{C}_{30,13}$	IWFDWWIMLFFILIDDWWLPWPWPHMMINGWWVWWWPHPFWMGWLYCPGC	$\mathcal{C}_{30,14}$	PNWFPPNLLPTSPLWPLCVPLLFFAWVPNFIWYMTVHWLYPPVSWLPHVC
$\mathcal{C}_{30,15}$	FIWWPGGAPWFWWWFWWWWAGPPWGWLFF	$\mathcal{C}_{30,16}$	LIWWIHHIWFTMTPHFHYHPGWWMVMIPHWCMWIICIAWAMYWWAIFHVL
$\mathcal{C}_{30,17}$	YAYVPPIASFNWGGGPFWWWPGWYSMVPYYHWMWCWHAWFIPYWMP	$\mathcal{C}_{30,18}$	GWMYPPIYPIWWITHYCPPGFYWTVLIWHLIMVIPIIGVCYYPGFIFVYA
$\mathcal{C}_{30,19}$	LWHFGTFGCPPLWVSPPRWGWPLFLIPWSGWHVLFGWPWPWYVWPRMY	$\mathcal{C}_{30,20}$	SCYRPPIFYFPWRRRPLWWVWWYWYTTGYIRWLWYHHAWWALSWPP
$\mathcal{C}_{30,21}$	PFWPCPIFVPPLFFPFLWPLPPLYTASFHWVPFFWFMFILHTLSYLTW	$\mathcal{C}_{30,22}$	RIFYFWWPWWHWWPWPFFRYPPFPYWAIW
$\mathcal{C}_{30,23}$	LWWNWYWWYFHYFWWNWHMNPHWFYWHWVHYYKYHWYFYRLKWWHWHHWR	$\mathcal{C}_{30,24}$	KWVWNWHRYFHFHVYWLFYWYWRHWYWPWHFFDIWFWLYLLPFYWNCYW
$\mathcal{C}_{30,25}$	LFSWFFWRHWRHWYMHKWHRVWVHHYDFFWFFWWHWWHWYHHWWHFWH	$\mathcal{C}_{30,26}$	DQWLFVDRWVWWRLWKWWKSKWLWWWWWWSWPWWWHWYCWYWPVWIYWWY
$\mathcal{C}_{30,27}$	DFFWINYGMWWWWVYSLVWWLWLPWVWWFYLYGYDFFWFFYFYWFHYW		
$\mathcal{C}_{31,1}$	HWWWNDWRDWWYHDVYFYIYNWKIWYSHYFWYYRWRNRWIKPWDRKYMPE	$\mathcal{C}_{31,2}$	EPFYMFYWDMYYYPDTYFYWHMWIMWCLFDVYYMHQWQITWFWKRKRKYK
$\mathcal{C}_{31,3}$	RWWWVIWHRYNWWRLDWYWSWPYHWYSWYHYWYDVWMPWIYYWDHFWCIS	$\mathcal{C}_{31,4}$	EWMWWKVWYYTDWWRWWRDFRCWWLRWWFRRHPYYLWWWYWYIWHWWHDR
$\mathcal{C}_{31,5}$	WYLLMNFVDWYYYWYYWMYIYHEYWYWPYWYWFPEFWCWRYYRWRWN	$\mathcal{C}_{31,6}$	WHWYYYVMDIHWDFCHWWWYFYEHHILPHKRWWHFWFMRMCLWHQFFWFHR
$\mathcal{C}_{31,7}$	TPSYCWVSSWWMFCQNYLHYDHWDHWYWFYRWWYQWWHRKKYFWWWWPWY	$\mathcal{C}_{31,8}$	HNFGWGWPYPMPHAWFVWIWWFGPMFGWWPASWGWHIFFPGPYSWHFSMW
$\mathcal{C}_{31,9}$	IVPPWFVHYVAIIIWMWRWPWLRIAWYFWCVAWIWHHYWKAWCKWMLWNP	$\mathcal{C}_{31,10}$	AWILIYIIYFCHLVHPWLSGHWWWWHHWAFCIHPCPPVFLLFFPHSTPHV
$\mathcal{C}_{31,11}$	DWWDYPMTWDWTMWFYSWWCVWFEYWEWWNWYCFWSLWLFFSIFWVHWW	$\mathcal{C}_{31,12}$	PTWACWPYPWWWFGLWAHVCHPYYWYIWFYLGGPYSPV
$\mathcal{C}_{31,13}$	WGWPIPWWWLWWWWWWAWWAW	$\mathcal{C}_{31,14}$	IWFDWWIMLFFILIDDWWLPWPWPHMMINGWWVWWWPHPFWMGWLYCPGC
$\mathcal{C}_{31,15}$	FIWWPGGAPWFWWWFWWWWAGPPWGWLFF	$\mathcal{C}_{31,16}$	LIWWIHHIWFTMTPHFHYHPGWWMVMIPHWCMWIICIAWAMYWWAIFHVL
$\mathcal{C}_{31,17}$	YAYVPPIASFNWGGGPFWWWPGWYSMVPYYHWMWCWHAWFIPYWMP	$\mathcal{C}_{31,18}$	GWMYPPIYPIWWITHYCPPGFYWTVLIWHLIMVIPIIGVCYYPGFIFVYA
$\mathcal{C}_{31,19}$	LWHFGTFGCPPLWVSPPRWGWPLFLIPWSGWHVLFGWPWPWYVWPRMY	$\mathcal{C}_{31,20}$	SCYRPPIFYFPWRRRPLWWVWWYWYTTGYIRWLWYHHAWWALSWPP
$\mathcal{C}_{31,21}$	PFWPCPIFVPPLFFPFLWPLPPLYTASFHWVPFFWFMFILHTLSYLTW	$\mathcal{C}_{31,22}$	GHWWPPHWPIWPPLPWPIWIPSFWWLPFFP
$\mathcal{C}_{31,23}$	RIFYFWWPWWHWWPWPFFRYPPFPYWAIW	$\mathcal{C}_{31,24}$	LWWNWYWWYFHYFWWNWHMNPHWFYWHWVHYYKYHWYFYRLKWWHWHHWR
$\mathcal{C}_{31,25}$	KWVWNWHRYFHFHVYWLFYWYWRHWYWPWHFFDIWFWLYLLPFYWNCYW	$\mathcal{C}_{31,26}$	LFSWFFWRHWRHWYMHKWHRVWVHHYDFFWFFWWHWWHWYHHWWHFWH
$\mathcal{C}_{31,27}$	DQWLFVDRWVWWRLWKWWKSKWLWWWWWWSWPWWWHWYCWYWPVWIYWWY	$\mathcal{C}_{31,28}$	DFFWINYGMWWWWVYSLVWWLWLPWVWWFYLYGYDFFWFFYFYWFHYW
$\mathcal{C}_{32,1}$	HWWWNDWRDWWYHDVYFYIYNWKIWYSHYFWYYRWRNRWIKPWDRKYMPE	$\mathcal{C}_{32,2}$	EPFYMFYWDMYYYPDTYFYWHMWIMWCLFDVYYMHQWQITWFWKRKRKYK
$\mathcal{C}_{32,3}$	RWWWVIWHRYNWWRLDWYWSWPYHWYSWYHYWYDVWMPWIYYWDHFWCIS	$\mathcal{C}_{32,4}$	EWMWWKVWYYTDWWRWWRDFRCWWLRWWFRRHPYYLWWWYWYIWHWWHDR

Table S6 continued:

$\mathcal{C}_{32,5}$	WYLLMNFVDWYYYWYYWMYIYHEYWYWPYWYWFPEFWCWRYYRWRWN	$\mathcal{C}_{32,6}$	WHWYYYVMDIHWDFCHWWWYFYEHHILPHKRWWHFWFMRMCLWHQFFWFHR
$\mathcal{C}_{32,7}$	TPSYCWVSSWWMFCQNYLHYDHWDHWYWFYRWWYQWWHRKKYFWWWWPWY	$\mathcal{C}_{32,8}$	HNFGWGWPYPMPHAWFVWIWWFGPMFGWWPASWGWHIFFPGPYSWHFSMW
$\mathcal{C}_{32,9}$	IVPPWFVHYVAIIIWMWRWPWLRIAWYFWCVAWIWHHYWKAWCKWMLWNP	$\mathcal{C}_{32,10}$	DWWDYPMTWDWTMWFYSWWCVWFEYWEWWNWYCFWSLWLFFSIFWVHWW
$\mathcal{C}_{32,11}$	PTWACWPYPWWWFGLWAHVCHPYYWYIWFYLGGPYSPV	$\mathcal{C}_{32,12}$	IWFDWWIMLFFILIDDWWLPWPWPHMMINGWWVWWWPHPFWMGWLYCPGC
$\mathcal{C}_{32,13}$	FIWWPGGAPWFWWWFWWWWAGPPWGWLFF	$\mathcal{C}_{32,14}$	LIWWIHHIWFTMTPHFHYHPGWWMVMIPHWCMWIICIAWAMYWWAIFHVL
$\mathcal{C}_{32,15}$	YAYVPPIASFNWGGGPFWWWPGWYSMVPYYHWMWCWHAWFIPYWMP	$\mathcal{C}_{32,16}$	LWHFGTFGCPPLWVSPPRWGWPLFLIPWSGWHVLFGWPWPWYVWPRMY
$\mathcal{C}_{32,17}$	SCYRPPIFYFPWRRRPLWWVWWYWYTTGYIRWLWYHHAWWALSWPP	$\mathcal{C}_{32,18}$	RIFYFWWPWWHWWPWPFFRYPPFPYWAIW
$\mathcal{C}_{32,19}$	LWWNWYWWYFHYFWWNWHMNPHWFYWHWVHYYKYHWYFYRLKWWHWHHWR	$\mathcal{C}_{32,20}$	KWVWNWHRYFHFHVYWLFYWYWRHWYWPWHFFDIWFWLYLLPFYWNCYW
$\mathcal{C}_{32,21}$	LFSWFFWRHWRHWYMHKWHRVWVHHYDFFWFFWWHWWHWYHHWWHFWH	$\mathcal{C}_{32,22}$	DQWLFVDRWVWWRLWKWWKSKWLWWWWWWSWPWWWHWYCWYWPVWIYWWY
$\mathcal{C}_{32,23}$	DFFWINYGMWWWWWVYSLVWWLWLPWVWWFYLYGYDFFWFFYFYWFHYW		
$\mathcal{C}_{33,1}$	HWWWNDWRDWWYHDVYFYIYNWKIWYSHYFWYYRWRNRWIKPWDRKYMPE	$\mathcal{C}_{33,2}$	EPFYMFYWDMYYYPDTYFYWHMWIMWCLFDVYYMHQWQITWFWKRKRKYK
$\mathcal{C}_{33,3}$	RWWWVIWHRYNWWRLDWYWSWPYHWYSWYHYWYDVWMPWIYYWDHFWCIS	$\mathcal{C}_{33,4}$	EWMWWKVWYYTDWWRWWRDFRCWWLRWWFRRHPYYLWWWYWYIWHWWHDR
$\mathcal{C}_{33,5}$	WYLLMNFVDWYYYWYYWMYIYHEYWYWPYWYWFPEFWCWRYYRWRWN	$\mathcal{C}_{33,6}$	WHWYYYVMDIHWDFCHWWWYFYEHHILPHKRWWHFWFMRMCLWHQFFWFHR
$\mathcal{C}_{33,7}$	TPSYCWVSSWWMFCQNYLHYDHWDHWYWFYRWWYQWWHRKKYFWWWWPWY	$\mathcal{C}_{33,8}$	HNFGWGWPYPMPHAWFVWIWWFGPMFGWWPASWGWHIFFPGPYSWHFSMW
$\mathcal{C}_{33,9}$	IVPPWFVHYVAIIIWMWRWPWLRIAWYFWCVAWIWHHYWKAWCKWMLWNP	$\mathcal{C}_{33,10}$	AWILIYIIYFCHLVHPWLSGHWWWWHHWAFCIHPCPPVFLLFFPHSTPHV
$\mathcal{C}_{33,11}$	DWWDYPMTWDWTMWFYSWWCVWFEYWEWWNWYCFWSLWLFFSIFWVHWW	$\mathcal{C}_{33,12}$	PTWACWPYPWWWFGLWAHVCHPYYWYIWFYLGGPYSPV
$\mathcal{C}_{33,13}$	IWFDWWIMLFFILIDDWWLPWPWPHMMINGWWVWWWPHPFWMGWLYCPGC	$\mathcal{C}_{33,14}$	FIWWPGGAPWFWWWFWWWWAGPPWGWLFF
$\mathcal{C}_{33,15}$	LIWWIHHIWFTMTPHFHYHPGWWMVMIPHWCMWIICIAWAMYWWAIFHVL	$\mathcal{C}_{33,16}$	YAYVPPIASFNWGGGPFWWWPGWYSMVPYYHWMWCWHAWFIPYWMP
$\mathcal{C}_{33,17}$	GWMYPPIYPIWWITHYCPPGFYWTVLIWHLIMVIPIIGVCYYPGFIFVYA	$\mathcal{C}_{33,18}$	LWHFGTFGCPPLWVSPPRWGWPLFLIPWSGWHVLFGWPWPWYVWPRMY
$\mathcal{C}_{33,19}$	SCYRPPIFYFPWRRRPLWWVWWYWYTTGYIRWLWYHHAWWALSWPP	$\mathcal{C}_{33,20}$	PFWPCPIFVPPLFFPFLWPLPPLYTASFHWVPFFWFMFILHTLSYLTW
$\mathcal{C}_{33,21}$	RIFYFWWPWWHWWPWPFFRYPPFPYWAIW	$\mathcal{C}_{33,22}$	LWWNWYWWYFHYFWWNWHMNPHWFYWHWVHYYKYHWYFYRLKWWHWHHWR
$\mathcal{C}_{33,23}$	KWVWNWHRYFHFHVYWLFYWYWRHWYWPWHFFDIWFWLYLLPFYWNCYW	$\mathcal{C}_{33,24}$	LFSWFFWRHWRHWYMHKWHRVWVHHYDFFWFFWWHWWHWYHHWWHFWH
$\mathcal{C}_{33,25}$	DQWLFVDRWVWWRLWKWWKSKWLWWWWWWSWPWWWHWYCWYWPVWIYWWY	$\mathcal{C}_{33,26}$	DFFWINYGMWWWWWVYSLVWWLWLPWVWWFYLYGYDFFWFFYFYWFHYW
$\mathcal{C}_{34,1}$	HWWWNDWRDWWYHDVYFYIYNWKIWYSHYFWYYRWRNRWIKPWDRKYMPE	$\mathcal{C}_{34,2}$	EPFYMFYWDMYYYPDTYFYWHMWIMWCLFDVYYMHQWQITWFWKRKRKYK
$\mathcal{C}_{34,3}$	RWWWVIWHRYNWWRLDWYWSWPYHWYSWYHYWYDVWMPWIYYWDHFWCIS	$\mathcal{C}_{34,4}$	EWMWWKVWYYTDWWRWWRDFRCWWLRWWFRRHPYYLWWWYWYIWHWWHDR
$\mathcal{C}_{34,5}$	WYLLMNFVDWYYYWYYWMYIYHEYWYWPYWYWFPEFWCWRYYRWRWN	$\mathcal{C}_{34,6}$	WHWYYYVMDIHWDFCHWWWYFYEHHILPHKRWWHFWFMRMCLWHQFFWFHR
$\mathcal{C}_{34,7}$	TPSYCWVSSWWMFCQNYLHYDHWDHWYWFYRWWYQWWHRKKYFWWWWPWY	$\mathcal{C}_{34,8}$	HNFGWGWPYPMPHAWFVWIWWFGPMFGWWPASWGWHIFFPGPYSWHFSMW
$\mathcal{C}_{34,9}$	IVPPWFVHYVAIIIWMWRWPWLRIAWYFWCVAWIWHHYWKAWCKWMLWNP	$\mathcal{C}_{34,10}$	AWILIYIIYFCHLVHPWLSGHWWWWHHWAFCIHPCPPVFLLFFPHSTPHV
$\mathcal{C}_{34,11}$	DWWDYPMTWDWTMWFYSWWCVWFEYWEWWNWYCFWSLWLFFSIFWVHWW	$\mathcal{C}_{34,12}$	PTWACWPYPWWWFGLWAHVCHPYYWYIWFYLGGPYSPV
$\mathcal{C}_{34,13}$	IWFDWWIMLFFILIDDWWLPWPWPHMMINGWWVWWWPHPFWMGWLYCPGC	$\mathcal{C}_{34,14}$	FIWWPGGAPWFWWWFWWWWAGPPWGWLFF
$\mathcal{C}_{34,15}$	LIWWIHHIWFTMTPHFHYHPGWWMVMIPHWCMWIICIAWAMYWWAIFHVL	$\mathcal{C}_{34,16}$	YAYVPPIASFNWGGGPFWWWPGWYSMVPYYHWMWCWHAWFIPYWMP
$\mathcal{C}_{34,17}$	LWHFGTFGCPPLWVSPPRWGWPLFLIPWSGWHVLFGWPWPWYVWPRMY	$\mathcal{C}_{34,18}$	SCYRPPIFYFPWRRRPLWWVWWYWYTTGYIRWLWYHHAWWALSWPP
$\mathcal{C}_{34,19}$	PFWPCPIFVPPLFFPFLWPLPPLYTASFHWVPFFWFMFILHTLSYLTW	$\mathcal{C}_{34,20}$	RIFYFWWPWWHWWPWPFFRYPPFPYWAIW
$\mathcal{C}_{34,21}$	LWWNWYWWYFHYFWWNWHMNPHWFYWHWVHYYKYHWYFYRLKWWHWHHWR	$\mathcal{C}_{34,22}$	KWVWNWHRYFHFHVYWLFYWYWRHWYWPWHFFDIWFWLYLLPFYWNCYW
$\mathcal{C}_{34,23}$	LFSWFFWRHWRHWYMHKWHRVWVHHYDFFWFFWWHWWHWYHHWWHFWH	$\mathcal{C}_{34,24}$	DQWLFVDRWVWWRLWKWWKSKWLWWWWWWSWPWWWHWYCWYWPVWIYWWY
$\mathcal{C}_{34,25}$	DFFWINYGMWWWWWVYSLVWWLWLPWVWWFYLYGYDFFWFFYFYWFHYW		
$\mathcal{C}_{35,1}$	HWWWNDWRDWWYHDVYFYIYNWKIWYSHYFWYYRWRNRWIKPWDRKYMPE	$\mathcal{C}_{35,2}$	EPFYMFYWDMYYYPDTYFYWHMWIMWCLFDVYYMHQWQITWFWKRKRKYK
$\mathcal{C}_{35,3}$	RWWWVIWHRYNWWRLDWYWSWPYHWYSWYHYWYDVWMPWIYYWDHFWCIS	$\mathcal{C}_{35,4}$	EWMWWKVWYYTDWWRWWRDFRCWWLRWWFRRHPYYLWWWYWYIWHWWHDR
$\mathcal{C}_{35,5}$	WYLLMNFVDWYYYWYYWMYIYHEYWYWPYWYWFPEFWCWRYYRWRWN	$\mathcal{C}_{35,6}$	WHWYYYVMDIHWDFCHWWWYFYEHHILPHKRWWHFWFMRMCLWHQFFWFHR
$\mathcal{C}_{35,7}$	TPSYCWVSSWWMFCQNYLHYDHWDHWYWFYRWWYQWWHRKKYFWWWWPWY	$\mathcal{C}_{35,8}$	HNFGWGWPYPMPHAWFVWIWWFGPMFGWWPASWGWHIFFPGPYSWHFSMW
$\mathcal{C}_{35,9}$	IVPPWFVHYVAIIIWMWRWPWLRIAWYFWCVAWIWHHYWKAWCKWMLWNP	$\mathcal{C}_{35,10}$	DWWDYPMTWDWTMWFYSWWCVWFEYWEWWNWYCFWSLWLFFSIFWVHWW
$\mathcal{C}_{35,11}$	PTWACWPYPWWWFGLWAHVCHPYYWYIWFYLGGPYSPV	$\mathcal{C}_{35,12}$	WGWPIPWWWLWWWWWWAWWAW
$\mathcal{C}_{35,13}$	IWFDWWIMLFFILIDDWWLPWPWPHMMINGWWVWWWPHPFWMGWLYCPGC	$\mathcal{C}_{35,14}$	FIWWPGGAPWFWWWFWWWWAGPPWGWLFF
$\mathcal{C}_{35,15}$	LIWWIHHIWFTMTPHFHYHPGWWMVMIPHWCMWIICIAWAMYWWAIFHVL	$\mathcal{C}_{35,16}$	YAYVPPIASFNWGGGPFWWWPGWYSMVPYYHWMWCWHAWFIPYWMP
$\mathcal{C}_{35,17}$	LWHFGTFGCPPLWVSPPRWGWPLFLIPWSGWHVLFGWPWPWYVWPRMY	$\mathcal{C}_{35,18}$	SCYRPPIFYFPWRRRPLWWVWWYWYTTGYIRWLWYHHAWWALSWPP
$\mathcal{C}_{35,19}$	PFWPCPIFVPPLFFPFLWPLPPLYTASFHWVPFFWFMFILHTLSYLTW	$\mathcal{C}_{35,20}$	GHWWPPHWPIWPPLPWPIWIPSFWWLPFFP
$\mathcal{C}_{35,21}$	RIFYFWWPWWHWWPWPFFRYPPFPYWAIW	$\mathcal{C}_{35,22}$	LWWNWYWWYFHYFWWNWHMNPHWFYWHWVHYYKYHWYFYRLKWWHWHHWR
$\mathcal{C}_{35,23}$	KWVWNWHRYFHFHVYWLFYWYWRHWYWPWHFFDIWFWLYLLPFYWNCYW	$\mathcal{C}_{35,24}$	LFSWFFWRHWRHWYMHKWHRVWVHHYDFFWFFWWHWWHWYHHWWHFWH
$\mathcal{C}_{35,25}$	DQWLFVDRWVWWRLWKWWKSKWLWWWWWWSWPWWWHWYCWYWPVWIYWWY	$\mathcal{C}_{35,26}$	DFFWINYGMWWWWWVYSLVWWLWLPWVWWFYLYGYDFFWFFYFYWFHYW

REFERENCES

1. C. P. Brangwynne, C. R. Eckmann, D. S. Courson, A. Rybarska, C. Hoege, J. Gharakhani, F. Jülicher, A. A. Hyman, Germline P granules are liquid droplets that localize by controlled dissolution/condensation. *Science* **324**, 1729–1732 (2009).
2. E. Gomes, J. Shorter, The molecular language of membraneless organelles. *J. Biol. Chem.* **294**, 7115–7127 (2019).
3. T. Mittag, R. V. Pappu, A conceptual framework for understanding phase separation and addressing open questions and challenges. *Mol. Cell* **82**, 2201–2214 (2022).
4. J.-Y. Kang, Z. Wen, D. Pan, Y. Zhang, Q. Li, A. Zhong, X. Yu, Y.-C. Wu, Y. Chen, X. Zhang, P.-C. Kou, J. Geng, Y.-Y. Wang, M.-M. Hua, R. Zong, B. Li, H.-J. Shi, D. Li, X.-D. Fu, J. Li, D. L. Nelson, X. Guo, Y. Zhou, L.-T. Gou, Y. Huang, M.-F. Liu, LLPS of FXR1 drives spermiogenesis by activating translation of stored mRNAs. *Science* **377**, eabj6647 (2022).
5. G. He, T. GrandPre, H. Wilson, Y. Zhang, M. C. Jonikas, N. S. Wingreen, Q. Wang, Phase-separating pyrenoid proteins form complexes in the dilute phase. *Commun. Biol.* **6**, 19 (2023).
6. Y. Shin, C. P. Brangwynne, Liquid phase condensation in cell physiology and disease. *Science* **357**, eaaf4382 (2017).
7. N.-N. Deng, W. T. Huck, Microfluidic formation of monodisperse coacervate organelles in liposomes. *Angew. Chem. Int. Ed. Engl.* **56**, 9736–9740 (2017).
8. T. Kojima, S. Takayama, Membraneless compartmentalization facilitates enzymatic cascade reactions and reduces substrate inhibition. *ACS Appl. Mater. Interfaces* **10**, 32782–32791 (2018).
9. M. K. Hazra, Y. Levy, Biophysics of phase separation of disordered proteins is governed by balance between short- and long-range interactions. *J. Phys. Chem. B* **125**, 2202–2211 (2021).
10. Y. G. Zhao, H. Zhang, Phase separation in membrane biology: The interplay between membrane-bound organelles and membraneless condensates. *Dev. Cell* **55**, 30–44 (2020).
11. I. Alshareedah, M. M. Moosa, M. Pham, D. A. Potoyan, P. R. Banerjee, Programmable viscoelasticity in protein-RNA condensates with disordered sticker-spacer polypeptides. *Nat. Commun.* **12**, 6620 (2021).
12. L. Cai, G. G. Wang, Through the lens of phase separation: Intrinsically unstructured protein and chromatin looping. *Nucleus* **14**, 2179766 (2023).

13. K. L. Price, M. Presler, C. M. Uyehara, D. C. Shakes. The intrinsically disordered protein spe-18 promotes localized assembly of MSP in *Caenorhabditis elegans* spermatocytes. *Development* **148**, dev195875 (2021).
14. L. Mediani, F. Antoniani, V. Galli, J. Vinet, A. D. Carra, I. Bigi, V. Tripathy, T. Tiago, M. Cimino, G. Leo, T. Amen, D. Kaganovich, C. Cereda, O. Pansarasa, J. Mandrioli, P. Tripathi, D. Troost, E. Aronica, J. Buchner, A. Goswami, J. Sterneckert, S. Alberti, S. Carra, Hsp90-mediated regulation of DYRK3 couples stress granule disassembly and growth via mTORC1 signaling. *EMBO Rep.* **22**, e51740 (2021).
15. A. Agarwal, S. K. Rai, A. Avni, S. Mukhopadhyay, An intrinsically disordered pathological prion variant Y145Stop converts into self-seeding amyloids via liquid–liquid phase separation. *Proc. Natl. Acad. Sci. U.S.A.* **118**, e2100968118 (2021).
16. M.-T. Wei, S. Elbaum-Garfinkle, A. S. Holehouse, C. C.-H. Chen, M. Feric, C. B. Arnold, R. D. Priestley, R. V. Pappu, C. P. Brangwynne, Phase behaviour of disordered proteins underlying low density and high permeability of liquid organelles. *Nat. Chem.* **9**, 1118–1125 (2017).
17. M. Feric, N. Vaidya, T. S. Harmon, D. M. Mitrea, L. Zhu, T. M. Richardson, R. W. Kriwacki, R. V. Pappu, C. P. Brangwynne, Coexisting liquid phases underlie nucleolar subcompartments. *Cell* **165**, 1686–1697 (2016).
18. G. L. Dignon, W. Zheng, R. B. Best, Y. C. Kim, J. Mittal, Relation between single-molecule properties and phase behavior of intrinsically disordered proteins. *Proc. Natl. Acad. Sci. U.S.A.* **115**, 9929–9934 (2018).
19. S. Das, A. N. Amin, Y.-H. Lin, H. S. Chan, Coarse-grained residue-based models of disordered protein condensates: Utility and limitations of simple charge pattern parameters. *Phys. Chem. Chem. Phys.* **20**, 28558–28574 (2018).
20. S. Das, Y.-H. Lin, R. M. Vernon, J. D. Forman-Kay, H. S. Chan, Comparative roles of charge, π, and hydrophobic interactions in sequence-dependent phase separation of intrinsically disordered proteins. *Proc. Natl. Acad. Sci. U.S.A.* **117**, 28795–28805 (2020), .
21. H. Y. J. Fung, M. Birol, E. Rhoades, IDPs in macromolecular complexes: The roles of multivalent interactions in diverse assemblies. *Curr. Opin. Struct. Biol.* **49**, 36–43 (2018).
22. S. E. Harding, P. Johnson, The concentration-dependence of macromolecular parameters. *Biochem. J.* **231**, 543–547 (1985).

23. R. Laghmach, I. Alshareedah, M. Pham, M. Raju, P. R. Banerjee, D. A. Potoyan, RNA chain length and stoichiometry govern surface tension and stability of protein-RNA condensates. *iScience* **25**, 104105 (2022).
24. G. M. Wadsworth, W. J. Zahurancik, X. Zeng, P. Pullara, L. B. Lai, V. Sidharthan, R. V. Pappu, V. Gopalan, P. R. Banerjee, RNAs undergo phase transitions with lower critical solution temperatures. bioRxiv 512593 [Preprint]. (2022). https://doi.org/10.1101/2022.10.17.512593.
25. C. J. Oldfield, A. K. Dunker, Intrinsically disordered proteins and intrinsically disordered protein regions. *Annu. Rev. Biochem.* **83**, 553–584 (2014).
26. R. M. Regy, J. Thompson, Y. C. Kim, J. Mittal, Improved coarse-grained model for studying sequence dependent phase separation of disordered proteins. *Protein Sci.* **30**, 1371–1379 (2021).
27. G. L. Dignon, W. Zheng, J. Mittal, Simulation methods for liquid–liquid phase separation of disordered proteins. *Curr. Opin. Chem. Eng.* **23**, 92–98 (2019).
28. G. L. Dignon, W. Zheng, Y. C. Kim, R. B. Best, J. Mittal, Sequence determinants of protein phase behavior from a coarse-grained model. *PLOS Comput. Biol.* **14**, e1005941 (2018).
29. J. A. Joseph, A. Reinhardt, A. Aguirre, P. Y. Chew, K. O. Russell, J. R. Espinosa, A. Garaizar, R. Collepardo-Guevara, Physics-driven coarse-grained model for biomolecular phase separation with near-quantitative accuracy. *Nat. Comput. Sci* **1**, 732–743 (2021).
30. M. A. Webb, N. E. Jackson, P. S. Gil, J. J. de Pablo, Targeted sequence design within the coarse-grained polymer genome. *Sci. Adv.* **6**, eabc6216 (2020).
31. A. J. Gormley, M. A. Webb, Machine learning in combinatorial polymer chemistry. *Nat. Rev. Mater.* **6**, 642–644 (2021).
32. J. S. Smith, B. Nebgen, N. Lubbers, O. Isayev, A. E. Roitberg, Less is more: Sampling chemical space with active learning. *J. Chem. Phys.* **148**, 241733 (2018).
33. C. Kim, A. Chandrasekaran, A. Jha, R. Ramprasad, Active-learning and materials design: The example of high glass transition temperature polymers. *MRS Commun.* **9**, 860–866 (2019).
34. B. Shahriari, K. Swersky, Z. Wang, R. P. Adams, N. De Freitas, Taking the human out of the loop: A review of Bayesian optimization. *Proc. IEEE* **104**, 148–175 (2016).
35. G. P. Wellawatte, A. Seshadri, A. D. White, Model agnostic generation of counterfactual explanations for molecules. *Chem. Sci.* **13**, 3697–3705 (2022).
36. G. P. Wellawatte, H. A. Gandhi, A. Seshadri, A. D. White, A perspective on explanations of molecular prediction models. *J. Chem. Theory Comput.* **19**, 2149–2160 (2023).

37. M. K. Hazra, Y. Levy, Affinity of disordered protein complexes is modulated by entropy-energy reinforcement. *Proc. Natl. Acad. Sci. U.S.A.* **119**, e2120456119 (2022).
38. D. A. McQuarrie, *Statistical Mechanics* (Harper Collins, 1976).
39. G. Vliegenthart, H. N. Lekkerkerker, Predicting the gas-liquid critical point from the second virial coefficient. *J. Chem. Phys.* **112**, 5364–5369 (2000).
40. R. Tuinier, G. A. Vliegenthart, H. N. Lekkerkerker, Depletion interaction between spheres immersed in a solution of ideal polymer chains. *J. Chem. Phys.* **113**, 10768–10775 (2000).
41. M. Rubinstein, R. H. Colby, *Polymer Physics* (Oxford Univ. Press, 2003), vol. 23.
42. S. Rekhi, D. S. Devarajan, M. P. Howard, Y. C. Kim, A. Nikoubashman, J. Mittal, Role of strong localized vs weak distributed interactions in disordered protein phase separation. *J. Phys. Chem. B* **127**, 3829–3838 (2023).
43. A. Z. Panagiotopoulos, Phase separation and aggregation in multiblock chains. *J. Chem. Phys.* **158**, 154901 (2023).
44. J. Comer, J. C. Gumbart, J. Hénin, T. Lelièvre, A. Pohorille, C. Chipot, The adaptive biasing force method: Everything you always wanted to know but were afraid to ask. *J. Phys. Chem. B* **119**, 1129–1151 (2015).
45. K. Binder, B. J. Block, P. Virnau, A. Tröster, Beyond the van der Waals loop: What can be learned from simulating Lennard-Jones fluids inside the region of phase coexistence. *Am. J. Phys* **80**, 1099–1109 (2012).
46. A. Hatos, B. Hajdu-Soltész, A. M. Monzon, N. Palopoli, L. Álvarez, B. Aykac-Fas, C. Bassot, G. I. Benítez, M. Bevilacqua, A. Chasapi, L. Chemes, N. E. Davey, R. Davidović, A Keith Dunker, A. Elofsson, J. Gobeill, N. S. González Foutel, G. Sudha, M. Guharoy, T. Horvath, V. Iglesias, A. V. Kajava, O. P. Kovacs, J. Lamb, M. Lambrughi, T. Lazar, J. Y. Leclercq, E. Leonardi, S. Macedo-Ribeiro, M. Macossay-Castillo, E. Maiani, J. A. Manso, C. Marino-Buslje, E. Martínez-Pérez, B. Mészáros, I. Mičetić, G. Minervini, N. Murvai, M. Necci, C. A. Ouzounis, M. Pajkos, L. Paladin, R. Pancsa, E. Papaleo, G. Parisi, E. Pasche, P. J. Barbosa Pereira, V. J. Promponas, J. Pujols, F. Quaglia, P. Ruch, M. Salvatore, E. Schad, B. Szabo, T. Szaniszló, S. Tamana, A. Tantos, N. Veljkovic, S. Ventura, W. Vranken, Z. Dosztányi, P. Tompa, S. C. E. Tosatto, D. Piovesan, DisProt: Intrinsic protein disorder annotation in 2020. *Nucleic Acids Res.* **48**, D269-D276 (2019).

47. J.-F. Yu, Z. Cao, Y. Yang, C.-L. Wang, Z.-D. Su, Y.-W. Zhao, J.-H. Wang, Y. Zhou, Natural protein sequences are more intrinsically disordered than random sequences. *Cell. Mol. Life Sci.* **73**, 2949–2957 (2016).
48. Q. Li, X. Peng, Y. Li, W. Tang, J. Zhu, J. Huang, Y. Qi, Z. Zhang, LLPSDB: A database of proteins undergoing liquid-liquid phase separation in vitro. *Nucleic Acids Res.* **48**, D320–D327 (2020).
49. K. Shmilovich, R. A. Mansbach, H. Sidky, O. E. Dunne, S. S. Panda, J. D. Tovar, A. L. Ferguson, Discovery of self-assembling π-conjugated peptides by active learning-directed coarse-grained molecular simulation. *J. Phys. Chem. B* **124**, 3873–3891 (2020).
50. M. J. Tamasi, R. A. Patel, C. H. Borca, S. Kosuri, H. Mugnier, R. Upadhya, N. S. Murthy, M. A. Webb, A. J. Gormley, Machine learning on a robotic platform for the design of polymer-protein hybrids. *Adv. Mater.* **34**, e2201809 (2022).
51. S. Kosuri, C. H. Borca, H. Mugnier, M. Tamasi, R. A. Patel, I. Perez, S. Kumar, Z. Finkel, R. Schloss, L. Cai, M. L. Yarmush, M. A. Webb, A. J. Gormley, Machine-assisted discovery of chondroitinase ABC complexes toward sustained neural regeneration. *Adv. Healthc. Mater.* **11**, e2102101 (2022).
52. K. M. Jablonka, G. M. Jothiappan, S. Wang, B. Smit, B. Yoo, Bias free multiobjective active learning for materials design and discovery. *Nat. Commun.* **12**, 2312 (2021).
53. A. Tran, J. Tranchida, T. Wildey, A. P. Thompson, Multi-fidelity machine-learning with uncertainty quantification and bayesian optimization for materials design: Application to ternary random alloys. *J. Chem. Phys.* **153**, 074705 (2020).
54. M. Emmerich, J.-W. Klinkenberg, The computation of the expected improvement in dominated hypervolume of Pareto front approximations. *Rapp. Tech. Leiden Univ.* **34**, 1–8 (2008).
55. M. Zuluaga, A. Krause, M. Püschel, ϵ-pal: An active learning approach to the multi-objective optimization problem. *J. Mach. Learn. Res.* **17**, 3619–3650 (2016).
56. K. Yang, M. Emmerich, A. Deutz, T. Bäck, Multi-objective bayesian global optimization using expected hypervolume improvement gradient, *Swarm Evol. Comput.* **44**, 945–956 (2019).
57. B. P. MacLeod, F. G. Parlane, C. C. Rupnow, K. E. Dettelbach, M. S. Elliott, T. D. Morrissey, T. H. Haley, O. Proskurin, M. B. Rooney, N. Taherimakhsousi, D. J. Dvorak, H. N. Chiu, C. E. B. Waizenegger, K. Ocean, M. Mokhtari, C. P. Berlinguette, A self-driving laboratory advances the Pareto front for material properties. *Nat. Commun.* **13**, 995 (2022).
58. R. A. Patel, C. H. Borca, M. A. Webb, Featurization strategies for polymer sequence or composition design by machine learning. *Mol. Syst. Des. Eng.* **7**, 661–676 (2022).

59. R. A. Patel, M. A. Webb, Data-driven design of polymer-based biomaterials: High-throughput simulation, experimentation, and machine learning. *ACS Appl. Bio Mater*. 10.1021/acsabm.2c00962 (2023).
60. W. Zheng, G. Dignon, M. Brown, Y. C. Kim, J. Mittal, Hydropathy patterning complements charge patterning to describe conformational preferences of disordered proteins. *J. Phys. Chem. Lett.* **11**, 3408–3415 (2020).
61. A. Statt, H. Casademunt, C. P. Brangwynne, A. Z. Panagiotopoulos, Model for disordered proteins with strongly sequence-dependent liquid phase behavior. *J. Chem. Phys.* **152**, 075101 (2020).
62. A. H. Mao, S. L. Crick, A. Vitalis, C. L. Chicoine, R. V. Pappu, Net charge per residue modulates conformational ensembles of intrinsically disordered proteins. *Proc. Natl. Acad. Sci. U.S.A.* **107**, 8183–8188 (2010).
63. I. Alshareedah, W. M. Borcherds, S. R. Cohen, M. Farag, A. Singh, A. Bremer, R. V. Pappu, T. Mittag, P. R. Banerjee, Sequence-encoded grammars determine material properties and physical aging of protein condensates. bioRxiv 535902 [Preprint] (2023). https://doi.org/10.1101/2023.04.06.535902.
64. S. Rekhi, C. G. Garcia, M. Barai, A. Rizuan, B. S. Schuster, K. L. Kiick, J. Mittal, Expanding the molecular language of protein liquid-liquid phase separation. bioRxiv 530853 [Preprint] (2023). https://doi.org/10.1101/2023.03.02.530853.
65. S. Wachter, B. Mittelstadt, C. Russell, Counterfactual explanations without opening the black box: Automated decisions and the gdpr. *Harv. JL & Tech.* **31**, 841–887 (2017).
66. S. Dandl, C. Molnar, M. Binder, B. Bischl, Multi-objective counterfactual explanations. arXiv:2004.11165 [stat.ML] (2020).
67. N. Salvi, A. Abyzov, M. Blackledge, Solvent-dependent segmental dynamics in intrinsically disordered proteins. *Sci. Adv.* **5**, eaax2348 (2019).
68. J. F. Rudzinski, Recent progress towards chemically-specific coarse-grained simulation models with consistent dynamical properties. *Comput. Secur.* **7**, 42 (2019).
69. S. Dhamankar, M. A. Webb, Chemically specific coarse-graining of polymers: Methods and prospects. *J. Poly. Sci.* **59**, 2613–2643 (2021).
70. A. Garaizar, J. R. Espinosa, Salt dependent phase behavior of intrinsically disordered proteins from a coarse-grained model with explicit water and ions. *J. Chem. Phys.* **155**, 125103 (2021).

71. A. P. Latham, B. Zhang, Maximum entropy optimized force field for intrinsically disordered proteins. *J. Chem. Theory Comput.* **16**, 773–781 (2019).
72. Z. Benayad, S. von Bülow, L. S. Stelzl, G. Hummer, Simulation of FUS protein condensates with an adapted coarse-grained model. *J. Chem. Theory Comput.* **17**, 525–537 (2020).
73. G. Tesei, T. K. Schulze, R. Crehuet, K. Lindorff-Larsen, Accurate model of liquid–liquid phase behavior of intrinsically disordered proteins from optimization of single-chain properties. *Proc. Natl. Acad. Sci. U.S.A.* **118**, e2111696118 (2021).
74. G. Tesei, K. Lindorff-Larsen, Improved predictions of phase behaviour of intrinsically disordered proteins by tuning the interaction range. *Open. Res. Europe* **2**, 94 (2022).
75. W. Zheng, G. L. Dignon, N. Jovic, X. Xu, R. M. Regy, N. L. Fawzi, Y. C. Kim, R. B. Best, J. Mittal, Molecular details of protein condensates probed by microsecond long atomistic simulations. *J. Phys. Chem. B* **124**, 11671–11679 (2020).
76. N. Galvanetto, M. T. Ivanović, A. Chowdhury, A. Sottini, M. Nüesch, D. Nettels, R. Best, B. Schuler, Ultrafast molecular dynamics observed within a dense protein condensate. bioRxiv 520135 [Preprint] (2022). https://doi.org/10.1101/2022.12.12.520135.
77. L. A. Gruijs da Silva, F. Simonetti, S. Hutten, H. Riemenschneider, E. L. Sternburg, L. M. Pietrek, J. Gebel, V. Dötsch, D. Edbauer, G. Hummer, L. S. Stelzl, D. Dormann, Disease-linked TDP-43 hyperphosphorylation suppresses TDP-43 condensation and aggregation. *EMBO J.* **41**, e108443 (2022).
78. W. M. Jacobs, Theory and simulation of multiphase coexistence in biomolecular mixtures. *J. Chem. Theory Comput.* **19**, 3429–3445 (2023).
79. S. Do, C. Lee, T. Lee, D.-N. Kim, Y. Shin, Engineering dna-based synthetic condensates with programmable material properties, compositions, and functionalities. *Sci. Adv.* **8**, eabj1771 (2022).
80. P. Y. Chew, J. A. Joseph, R. Collepardo-Guevara, A. Reinhardt, Physical determinants of multiphase organisation in multi-component protein/RNA condensates. *Biophys. J.* **122**, 295a–296a (2023).
81. A. P. Thompson, H. M. Aktulga, R. Berger, D. S. Bolintineanu, W. M. Brown, P. S. Crozier, P. J. in ‘t Veld, A. Kohlmeyer, S. G. Moore, T. D. Nguyen, R. Shan, M. J. Stevens, J. Tranchida, C. Trott, S. J. Plimpton, LAMMPS—A flexible simulation tool for particle-based materials modeling at the atomic, meso, and continuum scales. *Comput. Phys. Commun.* **271**, 108171 (2022).
82. C. E. Rasmussen, C. K. Williams, *Gaussian Processes for Machine Learning* (Springer, 2005), vol. 1.
83. G. Biau, E. Scornet, A random forest guided tour. *Test* **25**, 197–227 (2016).

84. M. Webb, W. Jacobs, Y. An, W. Oliver, *Thermodynamic and Dynamics Data for Coarse-Grained Intrinsically Disordered Proteins Generated by Active Learning* (Princeton University, 2023).
85. Y. Zhang, A. Otani, E. J. Maginn, Reliable viscosity calculation from equilibrium molecular dynamics simulations: A time decomposition method. *J. Chem. Theory Comput.* **11**, 3537–3546 (2015).
